# DNA methylation at a nutritionally sensitive region of the *PAX8* gene is associated with thyroid volume and function in Gambian children

**DOI:** 10.1126/sciadv.abj1561

**Published:** 2021-11-05

**Authors:** Toby Candler, Noah Kessler, Chathura Gunasekara, Kate Ward, Philip James, Eleonora Laritsky, Maria Baker, Roger Dyer, Rajavel Elango, David Jeffries, Robert Waterland, Sophie Moore, Marian Ludgate, Andrew Prentice, Matt Silver

**Affiliations:** 1 MRC Unit The Gambia at the London School of Hygiene and Tropical Medicine, London, UK.; 2 Department of Genetics, University of Cambridge, Cambridge, UK.; 3 USDA/ARS Children’s Nutrition Research Center, Department of Pediatrics, Baylor College of Medicine, Houston, TX, USA.; 4 MRC Lifecourse Epidemiology, University of Southampton, Southampton, UK.; 5 Department of Population Health, London School of Hygiene and Tropical Medicine, London, UK.; 6 British Columbia Children’s Hospital Research Institute, Vancouver, BC, Canada.; 7 Department of Molecular and Human Genetics, Baylor College of Medicine, Houston, TX, USA.; 8 Department of Women and Children’s Health, King’s College London, London, UK.; 9 Thyroid Research Group, School of Medicine, Cardiff University, Cardiff, UK.

## Abstract

PAX8 is a key thyroid transcription factor implicated in thyroid gland differentiation and function, and *PAX8* gene methylation is reported to be sensitive to the periconceptional environment. Using a novel recall-by-epigenotype study in Gambian children, we found that *PAX8* hypomethylation at age 2 years is associated with a 21% increase in thyroid volume and an increase in free thyroxine (T4) at 5 to 8 years, the latter equivalent to 8.4% of the normal range. Free T4 was associated with a decrease in DXA-derived body fat and bone mineral density. Furthermore, offspring *PAX8* methylation was associated with periconceptional maternal nutrition, and methylation variability was influenced by genotype, suggesting that sensitivity to environmental exposures may be under partial genetic control. Together, our results demonstrate a possible link between early environment, *PAX8* gene methylation and thyroid gland development and function, with potential implications for early embryonic programming of thyroid-related health and disease.

## Teaser

Child *PAX8* methylation is associated with thyroid volume and function and influenced by maternal periconceptional nutrition.

## INTRODUCTION

Thyroid hormones regulate a wide range of physiological processes and influence various outcomes related to cognition, growth, skeletal, cardiovascular, and metabolic health (*1*). While clinical sequelae of severe perturbations of thyroid hormone production are well documented (*2*), variation of thyroid function within the normal population reference range is also associated with a range of phenotypic traits including blood pressure, lipids, obesity, cardiovascular mortality, bone mineral density (BMD), and cancer risk (*3*).

The function of the hypothalamic-pituitary-thyroid axis is clinically assessed by measurement of pituitary-derived thyrotropin (TSH), free T4 (free thyroxine), and free T3 (free tri-iodothyronine). Higher TSH and lower free T4 are associated with adverse pregnancy outcomes (*3*) and increased body mass index (BMI) in adults (*4*) and children (*5*). Lower TSH and higher free T4 are associated with an increased risk of osteoporosis and fracture (*3*), and levels of free T4 are correlated with the concentration of various lipoproteins (*6*).

Heritability of thyroid function has been reported to be between 32 and 65% (free T4), 23 and 67% (free T3), and 32 and 65% (TSH) (*7*). However, current identified genetic variants associated with thyroid function contribute only a small amount to interindividual variation in thyroid hormone concentrations. For example, although a recent genome-wide association study (GWAS) identified 74 loci associated with TSH, together these explained just 13.3% of TSH variance, leaving much of the reported heritability unexplained (*8*). Moreover, congenital hypothyroidism (CHT), the most common endocrinopathy of childhood, is generally not inherited [less than 5% of cases have an identifiable genetic cause (*9*)] with 98% of cases nonfamilial (*10*) and a high discordance rate (92%) in monozygotic twins (*11*). In addition, seasonal variation in CHT incidence has been reported in the United Kingdom and Japan (*12*, *13*), and a recent study reported that prenatal famine exposure has been associated with higher TSH in adulthood (*14*). Together, these observations suggest that environmental factors and epigenetic or unknown genetic mechanisms may play a role in thyroid development and/or function.

PAX8 (paired-box 8) protein is one of four known thyroid transcription factors (TTFs) essential for thyroid development and function [others include NK2 homeobox 1 (NKX2-1), forkhead box E1 (FOXE1), and hematopoietically expressed homeobox (HHEX)] (*15*). These transcription factors regulate expression of thyroid-specific genes related to thyroid hormone production and storage such as TPO (thyroid peroxidase), Tg (thyroglobulin), and the sodium-iodide symporter (*15*, *16*) and are important for development and differentiation of the thyroid gland (*17*). PAX8 has been described as the “master regulator,” with a role in regulating the activity of other TTFs (*16*). *PAX8* knockout mice demonstrate that thyroid hypoplasia, low birth weight and growth retardation (*18*), and genetic mutations in *PAX8* can cause CHT in humans (*19*).

Epigenetic processes, including DNA methylation, histone modification, protein binding of DNA, chromatin remodeling, and RNA-based mechanisms, can affect gene expression (*20*). DNA methylation at CpG “islands” [cytosine (C) and guanine (G) separated by phosphate (p)] within promoter regions may regulate gene transcription in a tissue-specific manner (*21*). Promoter DNA methylation is usually associated with condensed heterochromatin and transcriptional down-regulation (*22*). Metastable epialleles (MEs) are epigenetic loci that demonstrate systemic (i.e., not tissue-specific) methylation with substantial variation between individuals (*23*). The methylation patterns are thought to be established early in embryonic development (*24*) and are not driven by genetic variation (*23*, *24*). Methylation at MEs is influenced by maternal diet around conception (*25*–*29*), and putative human MEs with stable methylation levels have been linked to disease-related phenotypes (*25*, *26*). These characteristics position MEs as potential epigenetic mediators of the effect of early environmental exposures in the developmental origins of health and disease (*30*).

The *PAX8* gene contains a putative human ME with evidence of systemic interindividual variation (*23*, *25*, *31*) and sensitivity to periconceptional and prenatal environment (see table S1). There is evidence that *PAX8* promoter methylation patterns in leucocytes and thyroid are concordant in children (*32*). Data from The Gambia showed that leucocyte *PAX8* methylation was higher in children conceived in the annual rainy (or “hungry”) season (*25*, *31*). In Bangladesh, higher methylation at the *PAX8* gene was reported in offspring following gestational famine exposure (*27*). A set of interlocking pathways, collectively known as one-carbon metabolism, provides methyl groups for DNA methylation and is dependent on multiple nutritional factors that act as substrates and essential cofactors (*33*). Seasonally driven variations in maternal circulating levels of one-carbon metabolites have been reported in The Gambia (*34*). Maternal supplementation with folic acid (a synthetic source of folate and a one-carbon metabolite) has been associated with differential *PAX8* methylation in adult offspring (*35*), and maternal preconception multiple micronutrient (MMN) supplementation was associated with differential methylation at *PAX8* in Gambian children (*36*).

Epigenetic influence on thyroid function or development has been little explored. Considering its key roles in thyroid development and regulation of the mature thyroid gland, and its epigenetic sensitivity to the early environment, *PAX8* is a prime candidate for study. Using a recall-by-epigenotype design, we examined links between *PAX8* DNA methylation measured at 2 years of age in peripheral blood and thyroid gland function and development in the same Gambian children aged 5 to 8 years. In addition, by examining body composition and BMD using dual energy x-ray absorptiometry (DXA) scans, we explored how *PAX8* methylation (via its putative effect on thyroid hormone production) may influence measures of adiposity and BMD. Using maternal biomarker data (including measures of one-carbon metabolites), we also explored how a child’s *PAX8* methylation status may be influenced by their mother’s nutritional status around the time of conception and assessed the influence of genetic variation in cis. Last, using data from The Cancer Genome Atlas (TCGA), we investigated the relationship between *PAX8* methylation and gene expression in thyroid tissue and assessed correlations between thyroid and whole blood methylation in samples from the Genotype-Tissue Expression (GTEx) Project.

## RESULTS

### PAX8 region of interest, participant selection, and baseline characteristics

**Fig. 1.**
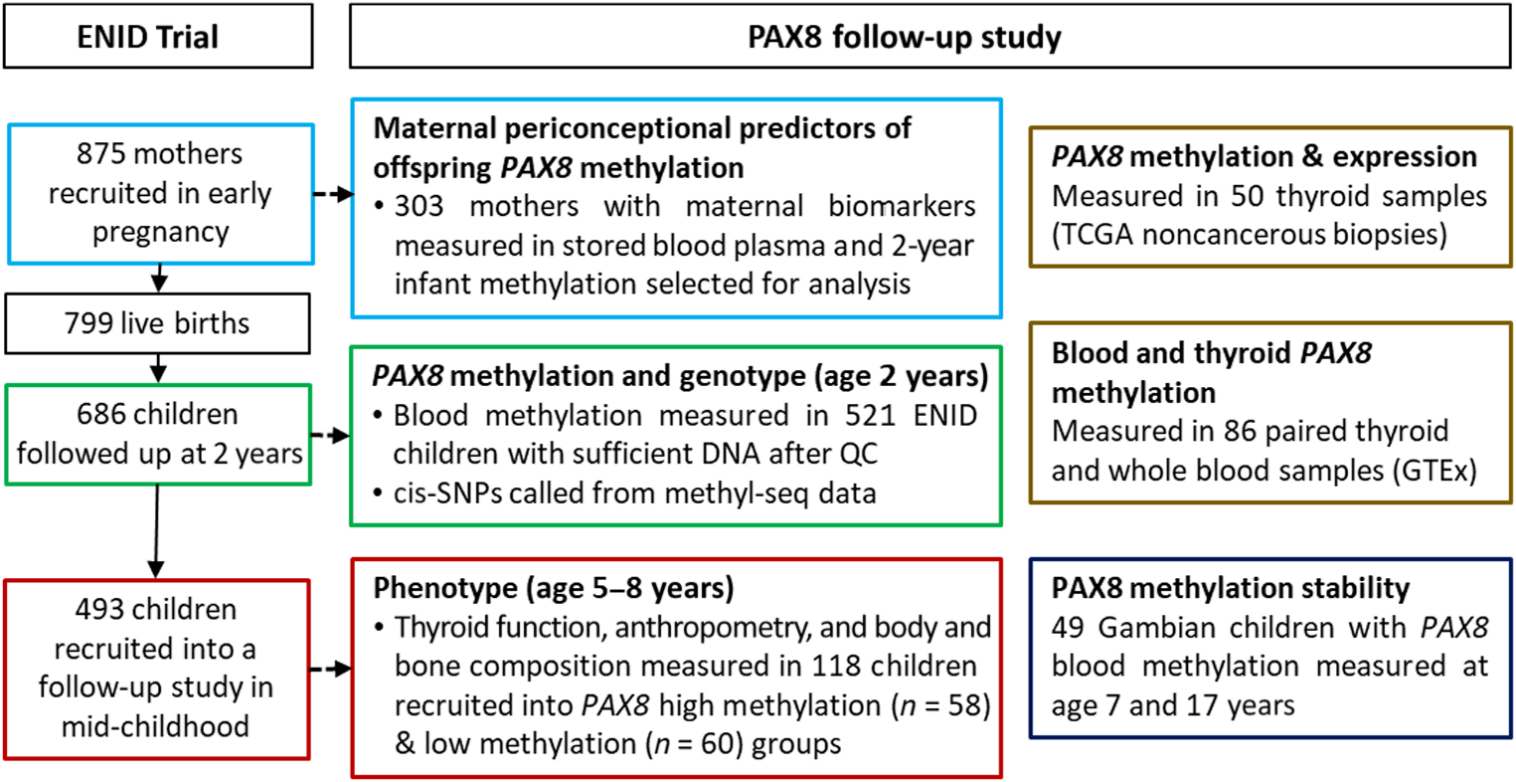
Study overview. QC, quality control.

**Fig. 2.**
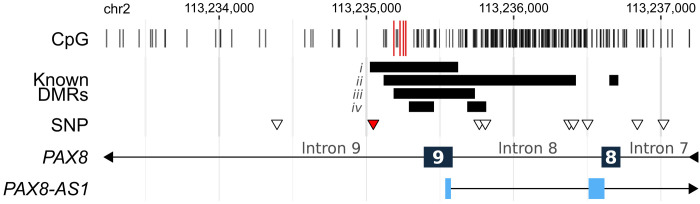
*PAX8* region of interest. The *PAX8* gene extends from chr2:113,215,997 to 113,278,921 (hg38) and contains 12 exons and 11 introns. Exons 8 and 9 of *PAX8* are shown, as well as the first two exons of an isoform of the *PAX8-AS1* antisense long noncoding RNA. CpGs are shown in the top track. The four CpGs highlighted in red were analyzed in this study (chr2:113,235,186; 113,235,228; 113,235,251; and 113,235,267). The “Known DMRs” (differentially methylated regions) track highlights regions identified in the following studies: putative MEs displaying systemic interindividual variation identified in (i) Silver *et al.* (*25*) and (ii) Kessler *et al.* (*23*); (iii) DMR associated with gestational famine (*27*), Gambian SoC (*35*), and maternal folic acid supplementation (*35*); and (iv) DMRs associated with Gambian SoC in an additional study (*31*). The SNP track denotes variants within 2000 bp of the CpGs of interest that were called from the methyl-seq data. The SNP highlighted in red is close to our region of interest and tags an LD block encompassing it (see fig. S11). This is the SNP used for the genotype analyses (rs10193733; chr2:113,235,047 T > C).

**Fig. 3.**
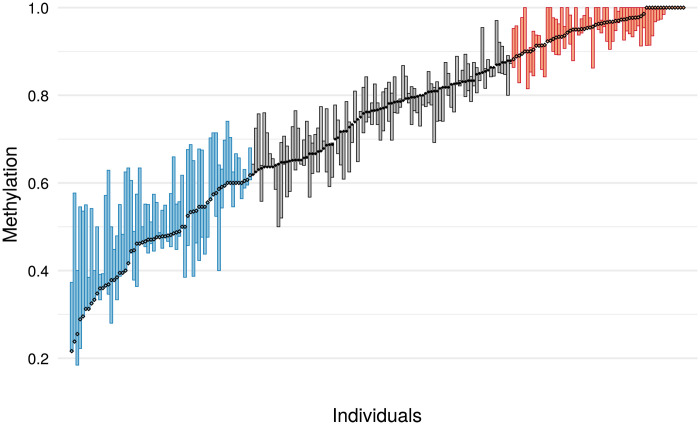
DNA methylation in the study population at the *PAX8* CpGs of interest. Individuals were sorted by methylation level at CpG chr2:113,235,228 (indicated by dots), which was used to select the high (red) and low (blue) *PAX8* methylation groups. Methylation range across the four CpGs of interest is indicated by the boxes.

Children from the “ENID” (Early Nutrition and Immune Development) trial (*37*) now aged between 5 and 8 years (*n* = 493) were recruited into “high” and “low” groups, according to their DNA methylation status in peripheral blood at age 2 years at a region of the *PAX8* gene (see Figs. 1 to 3). This region was selected on the basis of evidence of systemic interindividual variation and sensitivity to early environment from previous studies (*23*, *25*, *31*). DNA methylation was measured at four CpGs, which were highly correlated with adjacent CpGs and had sufficient coverage (see Materials and Methods and fig. S10 for further details).

One hundred eighteen children were recruited [high *PAX8* methylation group *n* = 58 (mean methylation (SD) = 0.96 (0.036)] and low *PAX8* methylation group *n* = 60 [0.50 (0.088)] with a median age [interquartile range (IQR)] of 7.18 years (1.67). Children in the low *PAX8* methylation group had a significantly lower BMI *z* score, but there were no significant differences in age, sex, weight-for-age *z* score (WAZ), height-for-age *z* score (HAZ), or pregnancy and infant supplementation received between the two groups (see Table 1).

**Table 1.** Baseline characteristics between high and low *PAX8* methylation groups. Group differences in normally distributed variables (WAZ, HAZ, and BMI) assessed by Student’s *t* test, nonnormally distributed variables (age) by Mann-Whitney *U* test, and categorical variables (sex and ENID supplementation group) by chi-square test. WAZ, weight-for-age *z* score; HAZ, height-for-age *z* score; PE, protein energy supplementation; FeFol, iron and folate supplementation; MMN, multiple micronutrient supplementation.

	**All (*n* = 118)**	**High *PAX8* methylation** **(*n* = 58)**	**Low *PAX8* methylation** **(*n* = 60)**	***P* value**
**Sex**				
Male	71	30	41	0.10
Female	47	28	19	
**Age (years)**				
Median [IQR]	7.18 [1.67]	7.28 [1.49]	7.09 [1.61]	0.29
Range	5.22 to 8.71	5.22 to 8.68	5.23 to 8.71	
Body size measures*				
Birth weight (kg) [SD]	3.03 [0.43]	3.01 [0.46]	3.05[0.41]	0.62
Mean WAZ [SD]	−1.25 [0.85]	−1.18 [0.87]	−1.32 [0.82]	0.36
Mean HAZ [SD]	−0.75 [0.83]	−0.81 [0.86]	−0.70 [0.79]	0.43
Mean BMI *z* score [SD]	−1.20 [0.87]	−1.01 [0.87]	−1.38 [0.84]	**0.02**
**ENID pregnancy supplementation**				
PE	31	16	15	0.90
FeFol	28	12	16	
MMN	26	13	13	
PE and MMN	33	17	16	
**ENID infant supplementation**				
MMN	54	27	27	1.00
No MMN	64	31	33	

*Body size measures reported were measured at recruitment for this current study (i.e., between 5 and 8 years of age).

### Associations between *PAX8* methylation and thyroid outcomes

#### Thyroid volume

Thyroid ultrasound scans were performed blinded to *PAX8* methylation group on 114 children (four children did not attend for scans). Of these, two had technical difficulties in ascertaining accurate measurements and were removed from subsequent analysis (both were in the high methylation group). A single case of right thyroid lobe hemiagenesis in the high *PAX8* methylation group was the only abnormality identified. This participant was retained in the analysis as total thyroid volume was within normal range.

In crude unadjusted analyses, total thyroid volume was elevated in the low methylation group (3.24 versus 2.87 cm^3^ in the high PAX8 methylation group, *P* = 0.035; see Table 2). In a multiple linear regression model adjusting for age, sex, BMI, and urinary iodine concentration (UIC), the association between *PAX8* methylation group and total thyroid volume was strengthened, yielding a 0.61 cm^3^ [SE = 0.15] or 21% higher thyroid volume in the low versus high group (*P* = 0.0001; see Table 3). This analysis also revealed significant associations between total thyroid volume and age, sex, BMI, and UIC (see table S2).

**Table 2.** Thyroid volume and function comparison by *PAX8* methylation group; crude (unadjusted) analyses. Group differences in normally distributed variables (thyroid volume, free T4, and free T3) were assessed by Student’s *t* test and nonnormally distributed variables (TSH and Tg) by Mann-Whitney *U* test.

	**All**	**High *PAX8* methylation**	**Low *PAX8* methylation**	***P* value**
**Thyroid volume**				
Mean total thyroid volume [SD] cm^3^	3.06 [0.93]	2.87 [0.83]	3.24 [0.98]	**0.035**
	*n* = 112	*n* = 53	*n* = 59	
**Thyroid function**				
Mean free T4 [SD], pM	13.6 [1.34]	13.3 [1.33]	13.9 [1.29]	**0.009**
	*n* = 114	*n* = 55	*n* = 59	
Mean free T3 [SD], pM	6.09 [0.68]	6.08 [0.63]	6.09 [0.74]	0.92
	*n* = 114	*n* = 55	*n* = 59	
Median TSH [IQR], mU/liter	1.82 [1.01]	1.86[0.92]	1.73 [1.08]	0.34
	*n* = 116	*n* = 56	*n* = 60	
Median Tg [IQR], μg/liter	19.2 [10.70]	18.5 [7.80]	19.4 [15.1]	0.46
	*n* = 105	*n* = 49	*n* = 56	

**Table 3.** *PAX8* methylation group as a predictor of thyroid volume and function; multiple linear regression (adjusted) analyses. Low *PAX8* methylation group coefficient gives the mean increase (decrease if negative) relative to the high *PAX8* methylation group. Adjustment covariates for the multiple linear regression model for total thyroid volume are age, sex, BMI, urinary iodine (see table S2). Adjustment covariates for multiple linear regression model for free T4, free T3, logTSH, and logTg are age, sex, and urinary iodine (see table S3).

**Outcome**	**Number of** **individuals**	**Low *PAX8*** **methylation** **group** **coefficient [SE]**	***P* value**
Total Thyroid Volume (cm^3^)	112	0.61 [0.15]	**0.0001**
Free T4 (pM)	113	0.85 [0.24]	**0.0007**
Free T3 (pM)	113	−0.02 [0.13]	0.88
Log TSH (mU/ liter)	115	−0.11 [0.09]	0.26
Log Tg (μg/liter)	94	0.02 [0.12]	0.85

#### Thyroid function

In crude comparisons of thyroid function measures (free T4, free T3, TSH, and Tg) between the two groups, free T4 was observed to be significantly lower in the high *PAX8* methylation group (13.3 versus 13.9 pM, *P* = 0.009; see Table 2).

Iodine insufficiency was evaluated by two methods: plasma Tg level and UIC. On the basis of Tg level, there was no difference in iodine insufficiency between the groups, but significantly more children were classified with iodine insufficiency by UIC in the high *PAX8* methylation group compared to the low *PAX8* methylation group [21 of 56 (37.5%) versus 10 of 60 (16.7%), *P* = 0.02], and UIC was also higher in the low *PAX8* methylation group (170 versus 128 μg/liter, *P* = 0.04; see table S3). Since iodine concentration and/or insufficiency can affect thyroid function, UIC was included as an adjustment covariate in regression models to ensure appropriate adjustment was made for these group differences.

In a multiple linear regression model with adjustment for age, sex, and UIC, free T4 level was 0.85 pM [SE = 0.24] higher in the low *PAX8* methylation group (*P* = 0.0007; see Table 3 and table S4). The laboratory normal range for free T4 is between 9.0 and 19.1 pM, so this difference between the two groups—8.4% of the normal range—is substantial. No significant associations between *PAX8* methylation and TSH, free T3, or Tg were observed after adjustment for age, sex, and UIC (see Table 3 and table S4).

### Association between free T4 and body composition and bone mineral outcomes

We hypothesized that *PAX8* methylation (via its putative effect on thyroid hormone production) may influence measures of adiposity and BMD. To explore potential associations between free T4 and fat mass index (FMI), lean mass index (LMI), and BMD, whole-body DXA scans were performed on 113 of the 5- to 8-year-old children.

In multiple regression models adjusted for relevant covariates, log FMI was associated with free T4 (β = −0.04 [SE 0.02], *P* = 0.033; see Table 4 and table S5), so that for every picomolar increase in free T4, there was a 4.3% reduction in FMI. Free T4 was not associated with LMI (see table S6). Free T4 was inversely associated with log total-body-less-head (TBLH) BMD (β = −0.008 [0.004], *P* = 0.044), so that TBLH BMD was reduced by 0.8% for every picomolar increase in free T4 (see Table 4 and table S7).

**Table 4.** Free T4 as a predictor of FMI and BMD; multiple linear regression (adjusted) analyses. Adjustment covariates for the multiple linear regression model for logFMI are age, sex, and weight (see table S4). Adjustment covariates for multiple linear regression models for log TBLH BMD and log TBLH BMC are age, sex, height, and weight (see table S6).

**Outcome**	**Number of** **individuals**	**Free T4** **(pM)** **coefficient** **[SE]**	**% change** **in outcome** **per one** **unit** **increase in** free T4*	***P* value**
Log FMI [fat mass (kg)/m^2^]	113	−0.04 [0.02]	−4.30	**0.033**
Log TBLH BMD (g/cm^2^)	113	−0.008 [0.004]	−0.80	**0.044**
		112		114

*Back transformation if outcome log-transformed using [exp[β] − 1] × 100.

### Causal mediation analysis

Since we demonstrated significant associations between *PAX8* methylation group and both thyroid volume and free T4, we postulated that the methylation state set early in embryonic development (which we measured at the age of 2) could influence thyroid development and volume via regulation of *PAX8* expression and that this change in thyroid volume could in turn affect thyroid function and free T4 measured at 5 to 7 years of age. We therefore conducted a causal mediation analysis to test this, but this provided no evidence that the effect of differential methylation at PAX8 on thyroid function was mediated by thyroid volume [average causal mediation effect (ACME) or mediated effect = 0.031, 95% confidence interval −0.15 to 0.22, *P* = 0.74; see fig. S1]. As we did not find any evidence of a significant effect of *PAX8* methylation group on either BMD or any fat measure, we did not perform causal mediation analysis for these pathways.

### Predictors of *PAX8* methylation

We performed an analysis of the potential influence of sex and child and maternal BMI on *PAX8* methylation in *n* = 521 ENID-recruited children with 2-year *PAX8* methylation measurements (i.e., not restricted to the *n* = 118 high and low methylation groups analyzed above). In simple linear regression models, sex was significantly associated with *PAX8* methylation, with males having 0.24 [SE = 0.09] lower mean logit methylation compared to females (*P* = 0.005; see fig. S2 for comparison of equivalent untransformed mean % methylation difference). There were no significant associations between *PAX8* methylation and child BMI *z* score (β = −0.02 [SE = 0.04], *P* = 0.64), maternal BMI (β = 0.0004 [0.01], *P* = 0.98), infant WAZ (β = −0.05 [0.05], *P* = 0.27), or season of conception (SoC) (β = 0.09 [0.09], *P* = 0.29).

Three hundred three of the 521 children with *PAX8* methylation data also had paired maternal biomarker data. In multiple linear regression models adjusted for sex, four one-carbon metabolites were associated with a decrease in *PAX8* methylation (see Table 5): homocysteine (standardized β = −0.11 [0.05], *P* = 0.048), cysteine (β = −0.16 [0.05], *P* = 0.003), vitamin B12 (β = −0.1 [0.05], *P* = 0.05), and vitamin B6/pyridoxal-5′-phosphate (PLP, a vitamin B6 vitamer) (β = −0.12 [0.06], *P* = 0.03).

**Table 5.** Maternal one-carbon metabolite biomarkers as predictors of *PAX8* Methylation. Multiple linear regression models have *PAX8* mean logit methylation *z* score as the dependent variable with maternal one-carbon metabolites measured in maternal plasma and back-extrapolated to the time of conception as predictors, adjusted for sex. Hcy, homocysteine; DMG, dimethylglycine; PLP, pyridoxal 5-phosphate (B6 vitamer).

**Covariate**	**Standardized coefficient**	**SE**	***t* value**	***P* value**
**Hcy (μM)**	−0.11	0.05	−1.99	**0.048**
**Methionine (μM)**	−0.01	0.06	−0.13	0.89
**Cysteine (μM)**	−0.16	0.05	−2.95	**0.003**
**Choline (μM)**	−0.01	0.06	−0.20	0.84
**Betaine (μM)**	−0.07	0.05	−1.29	0.20
**DMG (μM)**	−0.03	0.06	0.52	0.61
**B12 (pM)**	−0.10	0.05	−1.96	**0.050**
**Folate (nM)**	−0.07	0.05	−1.37	0.17
**PLP (nM)**	−0.12	0.06	−2.17	**0.031**
**Riboflavin (nM)**	−0.11	0.06	−1.93	0.055

We also tested an extended panel of maternal biomarkers including amino acid levels (see table S8). Two of these biomarkers were associated with an increase in *PAX8* methylation: uracil (standardized β = 0.21 [0.05], *P* = 0.0001) and arginine (β = 0.16 [0.06], *P* = 0.006). Valine was associated with a decrease in *PAX8* methylation (β = −0.13 [0.05], *P* = 0.02).

### *PAX8* methylation, gene expression, and methylation tissue concordance

We next investigated the relationship between *PAX8* methylation and gene expression in thyroid tissue by using data from TCGA. TCGA provides gene expression and DNA methylation data on a variety of noncancerous tissues including 50 thyroid samples. The methylation data are derived from the Illumina HM450 array, which includes two of the *PAX8* CpGs of interest in this study. Since this region overlaps transcripts from sense (*PAX8*) and antisense (*PAX8-AS1*) genes, we considered the expression of both genes. Using fragments per kilobase per million mapped reads (FPKM) as an expression metric, *PAX8* and *PAX8-AS1* expression were positively correlated (Spearman *R* = 0.477, *P* = 0.0006; see fig. S3, left), but we found only weak evidence of an inverse relationship between *PAX8* methylation and *PAX8-AS1* or *PAX8* expression (see fig. S4, top). However, with RNA sequencing by expectation maximization (RSEM) as the metric of gene expression, we found strong evidence that *PAX8* methylation was negatively correlated with *PAX8-AS1* expression (Spearman *R* = −0.70, *P* = 6 × 10^−8^; see fig. S4, bottom right), although there was no correlation between *PAX8* and *PAX8-AS1* expression (fig. S3, right). There was no association between *PAX8* methylation and expression using RSEM (fig. S4, bottom left).

We analyzed mean DNA methylation across the four CpGs of interest in paired peripheral blood cell and thyroid tissue from 86 adult samples from the GTEx Project (*38*) and found no correlation (see fig. S5). Public GTEx expression data from 54 tissues shows that both *PAX8* and *PAX8-AS1* are predominantly expressed in thyroid (see fig. S6). Note that, in both the TGCA and GTEx dataset, thyroid methylation levels of the *PAX8* region of interest fall only within the high methylation group from our Gambian analysis in peripheral blood. Together, our interpretation is that although individual variation in PAX8 methylation in the early embryo may affect thyroid development, at some stage of thyroid development, this region becomes uniformly highly methylated in thyroid of all individuals.

### Stability of *PAX8* methylation from 7 to 17 years of age

We measured *PAX8* methylation in 49 Gambian children from stored peripheral blood DNA taken at age 7 and 17 years by pyrosequencing. Methylation was highly correlated between the two age groups (*R* = 0.76, *P* < 2.2 × 10^−16^; see fig. S7), indicating that blood cell methylation at this locus is highly stable across this age range.

### Exploring the relationship between *PAX8* methylation, genotype, and thyroid function

**Fig. 4.**
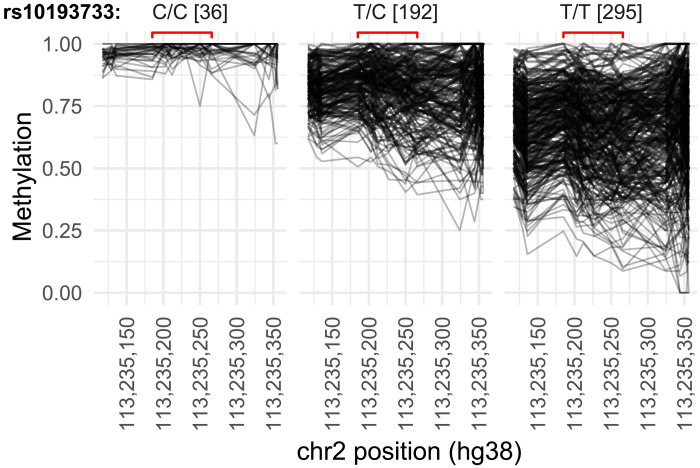
C alleles at rs10193733 are associated with decreased DNA methylation variation at the *PAX8* region of interest. DNA methylation at CpGs is shown for the *PAX8* region of interest (delimited by red brackets) and flanking 200-bp region, split by rs10193733 genotype. Numbers in brackets denote the counts of individuals with each genotype at the SNP (total *N* = 523).

We focused on a single-nucleotide polymorphism (SNP) (rs10193733) proximal to our *PAX8* region of interest for genetic analyses (see Fig. 2 and Materials and Methods for rationale for selecting this SNP). We observed higher methylation for homozygotes for the alternate allele (C/C, mean methylation = 0.97) compared to heterozygotes (T/C = 0.82 or wild-type alleles (T/T = 0.66; see Fig. 4). However, the most notable was that homozygotes for the alternate allele had a markedly limited methylation range (C/C, range = 0.93 to 1.00) compared to heterozygotes (C/T, range = 0.50 to 0.99) or homozygotes for the reference allele (T/T, range = 0.18 to 1.00; see Fig. 4).

This led us to postulate that the genotype with the greatest variability (T/T) could be more sensitive to periconceptional environment, offering a potential explanation for the lack of evidence for a SoC effect at this locus. Methylation distributions stratified by genotype suggested a potential interaction between SoC and genotype (see fig. S8), although this was not statistically significant, possibly due to a lack of power. We also found no evidence for an interaction between maternal nutritional biomarkers and genotype.

We next assessed the direct effect of rs10193733 genotype on free T4 and total thyroid volume in multiple linear regression models with free T4 and total thyroid volume as the dependent variables regressed against the number of rs10193733 C alleles and adjusted for age, sex, and UIC. The C allele had a significant effect on both free T4 (β (per C allele) = −0.61 [SE = 0.16], *P* = 0.0002) and thyroid volume [β (per C allele) = −0.25 [0.11], *P* = 0.02]. As expected, (see Figs. 3 and 4), rs10193733 genotype was also significantly associated with *PAX8* methylation group (chi-square test *P* = 4.24 × 10^−13^). However, the effect of *PAX8* methylation group on thyroid volume and free T4 did not appear to be purely driven by genotype, as T/C and T/T individuals were well represented in both the high and low PAX8 methylation groups (see fig. S9).

## DISCUSSION

Previous studies in The Gambia and elsewhere have shown a consistent association between DNA methylation at a genomic region in the *PAX8* gene and the maternal periconceptional environment (*25*, *27*, *29*, *31*). Here, we have demonstrated an association between *PAX8* methylation and thyroid function in Gambian children, specifically free T4 and thyroid volume, with a relatively large effect size. Thyroid volume differed by >20% between low and high *PAX8* methylation groups after adjusting for covariates. Free T4 demonstrated a difference of 0.85 pM between groups or 8.4% of the normal range.

A recent GWAS of thyroid function found four SNPs [with minor allele frequency (MAF) > 1%] associated with free T4. The largest reported SNP effect was 0.22 pM per variant allele, and overall, common genetic variants together explained just more than 20% of the variance in free T4 (*39*). The difference in free T4 between high and low *PAX8* methylation groups in our study is therefore much greater than the largest individual genetic effect previously observed (0.85 versus 0.22 pM). Similarly, a recent GWAS found four variants associated with thyroid volume explaining just more than 3% of thyroid volume variance (*40*). Here, the largest SNP effect size was 0.093 cm^3^ per allele, again far smaller than the effect size seen in our study (0.61 cm^3^ after adjustment for covariates).

Few studies have analyzed associations between DNA methylation and thyroid function. A recent epigenome-wide association study (EWAS) did not find any associations with free T4 but did identify two differentially methylated positions (DMPs) associated with TSH and six DMPs associated with free T3 (*41*). There were no DMPs from the *PAX8* gene associated with thyroid function in this EWAS, although the study analyzed data from older European children (aged 14 to 17 years), and they may not have analyzed CpGs from our region of interest that has limited coverage on the Illumina HM450k array used in the study.

Our previous work in The Gambia demonstrated that *PAX8* methylation is sensitive to the periconceptional environment, with rainy season conceptions having higher methylation compared to conceptions in the dry season (*25*, *31*). There is evidence to suggest that the prenatal window where environmental exposures could influence *PAX8* gene methylation may not be limited to the periconceptional period. Famine exposure (for at least 7 months prenatally) (*27*) and pregnancy folic acid supplementation (*35*) have been associated with *PAX8* gene methylation in offspring. We did not find a significant SoC effect in this cohort. Our CpGs of interest only partially overlap the SoC-sensitive *PAX8* differentially methylated region highlighted by Silver *et al*. (*25*), and they are adjacent to (but do not include) the SoC-sensitive region reported by Waterland *et al*. (*31*). Thus, while they are correlated with regions previously identified as being sensitive to SoC effects (see fig. S10), the *PAX8* CpGs in this study were different and may be less sensitive to SoC effects. In addition, seasonal effects vary from year to year [see figure S12 by Waterland *et al.* (*31*)], so that it is possible that there may have been a diminished seasonal effect during the period of ENID trial recruitment compared to previous years. However, we did find associations with circulating levels of several one-carbon metabolites and amino acids measured in maternal plasma and back-extrapolated to the time of conception, and these have previously been shown to vary seasonally in this population (*34*). One-carbon metabolites covary through complex interactions so that, as previously, we have presented their nominal associations, unadjusted for multiple comparisons (*26*, *33*, *42*).

There is evidence that the *PAX8* region of interest is an ME (*23*, *25*, *27*), and circulating levels of one-carbon metabolites at the time of conception have previously been associated with offspring DNA methylation at several MEs in our population (*25*, *26*, *33*). In our study, increased cysteine, homocysteine, and PLP (a B6 vitamer) were associated with lower DNA methylation at the *PAX8* region of interest, as previously observed at other MEs (*29*, *42*). Furthermore, we identified the amino acids arginine and valine as potential predictors of *PAX8* methylation in a wider panel of periconceptional amino acid data. Amino acids and one-carbon metabolites are bidirectionally linked: Amino acids can supply cells with one-carbon metabolites (*43*), and levels of one-carbon metabolites can influence amino acid metabolism (*44*).

We observed a strong effect of genotype on DNA methylation variability, raising the possibility of a genotype-early environment interaction effect on methylation, as has been observed in a number of studies (*45*, *46*). We did not find evidence for a genotype-SoC interaction at the *PAX8* region studied, although we may have had limited power to detect this. We noted the presence of an indel polymorphism within the *PAX8* variably methylated region and in very strong linkage disequilibrium (LD) with the cis-SNP associated with *PAX8* methylation variability. It is plausible that this polymorphism influences the binding of a protein that affects methylation variability. Future work should explore this and other mechanisms underpinning the strong variability effect that we observed and its potential link to differential sensitivity to environmental factors.

**Fig. 5.**
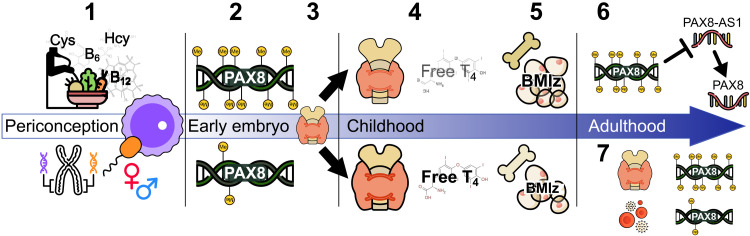
Proposed model linking *PAX8* methylation and expression, thyroid function and development, and phenotype. (1) *PAX8* methylation is influenced by periconceptional nutritional factors, sex, and genotype. (2) *PAX8* methylation is set early in embryonic development. (3) PAX8 methylation sets a trajectory for thyroid gland development: *PAX8* is expressed from gestational days 20 to 22 in humans, around the same time that thyroid progenitor cells begin specification in the endoderm (*15*). *PAX8* methylation state (or related epigenetic factors) alters *PAX8* expression that influences thyroid gland development. (4) *PAX8* methylation is inversely associated with thyroid size and free T4 in mid-childhood. (5) Free T4 is inversely associated with fat and BMD in mid-childhood. (6) *PAX8* methylation is inversely associated with *PAX8-AS1* expression. (7) There is a divergence between blood and thyroid methylation in adults.

In this study, *PAX8* methylation was measured at 2 years of age in peripheral blood, with phenotypic measures of thyroid function measured at 5 to 8 years. There is evidence that methylation in this region is systemic, with similar methylation patterns across tissues derived from endoderm, ectoderm, and mesoderm lineages, suggesting that methylation is established before gastrulation (*23*, *29*, *31*, *47*). We therefore speculate that thyroid *PAX8* methylation, influenced by environmental factors and established early in gestation, sets a trajectory for thyroid gland development that is reflected in differential thyroid morphology and function in mid-childhood (see Fig. 5).

*PAX8* is expressed early in embryological development [from days 20 to 22 in humans (*15*)]. Therefore, methylation patterns established in the cleavage-stage embryo could influence *PAX8* gene expression from the beginning of thyroid growth and development. Furthermore, methylation-driven differential *PAX8* expression in the differentiated gland could explain the difference in free T4 between the groups if high *PAX8* methylation down-regulates thyroid-specific genes associated with free T4 production. Further work is required to elucidate the cellular and molecular mechanisms underpinning these relationships.

Although our finding that this region is highly methylated in adult thyroid tissue from GTEx and TCGA appears to contradict this model, it is possible that thyroid methylation follows the systemic pattern in gestation and early life, followed by aging-associated hypermethylation specifically in thyroid. This is consistent with a previous study in children that compared DNA methylation in the *PAX8* promoter between leucocytes and thyroid tissue and found them to be correlated (*32*). A similar lineage-specific effect was observed at the murine AxinFused ME (*48*).

Our work also highlights a potential epigenetic contribution to thyroid gland development that has implications for understanding the etiology of nonheritable thyroid pathologies such as CHT. CHT is one of the top treatable causes of neurodevelopmental delay and is the commonest endocrinopathy of childhood (incidence between 1:2000 and 1:4000 newborns) (*49*); however, only a small proportion of CHT cases are attributed to known genetic mutations. Previous studies in Europe and Asia have found that the incidence of CHT displays a seasonal pattern (*12*, *13*), in support of an environmental component in the etiology of CHT. Further characterization of environmentally sensitive epigenetic regions (such as *PAX8*) associated with thyroid development could thus have public health implications in identifying environmental drivers for CHT. Sexually dimorphic methylation effects such as those we observed at *PAX8* [and in a previous Gambian study (*29*)] may also help to explain the preponderance for CHT in females (2:1) (*49*).

We identified one child from the high methylation group with thyroid hemiagenesis, a rare developmental thyroid anomaly (prevalence reported to be between 0.05 and 0.5%) that has been associated with *PAX8* gene mutations (*50*). While we cannot attribute *PAX8* hypermethylation as the direct cause of this anomaly, the possible contribution of epigenetic alterations to abnormal thyroid development warrants further research.

In this study, we have also demonstrated associations between free T4 and body fat and BMD in Gambian children. In common with our findings, there is growing evidence that free T4 levels within the normal range are inversely correlated with measures of body fat (*51*). Data from Korean children found that free T4 was inversely correlated with waist circumference, BMI, and markers of insulin resistance (*51*). A study in U.K. children demonstrated that free T4 was negatively associated with FMI (from DXA) and BMI. To explore the direction of causation, the authors used Mendelian randomization and found that while BMI and body fat caused an increase in free T3, they did not seem to influence levels of free T4 (*5*). Thyroid volume and TSH have been shown to change in response to weight loss, although free T4 remains unchanged, further supporting the idea that free T4 is not influenced by body composition (*52*). Data from African children are limited, although a positive correlation between free T3/T4 ratio and BMI has been reported in Nigerian children (*53*). We found no association between free T4 and measures of lean mass, although an inverse relationship between free T4 and lean mass has been reported previously (*54*).

Free T4 has been reported to be negatively associated with BMD in adults (*3*), and higher free T4 (within the population reference range) is associated with an increased incidence of fractures in older people (*55*). Chondrocytes, osteoblasts, and osteoclasts express TSH receptor and thyroid hormone receptor, and thus, there is a biological target for thyroid hormone to affect bone modeling (*56*). Our novel finding that BMD is associated with free T4 in mid-childhood is of great interest as this reflects an important period of bone accrual and attainment of peak bone mass, which is related to future fracture risk. We found no overall effect of *PAX8* methylation on BMD and so did not investigate the possibility that the effect of methylation on BMD is mediated by free T4. However, it is possible that *PAX8* methylation may exert countervailing effects on BMD such that the net effect is nonsignificant despite their being a significant causal pathway between *PAX8* methylation, free T4, and BMD. *PAX8* has been reported to be hypomethylated in human cartilage in individuals with osteoarthritis (*57*), which is of interest considering our finding that low *PAX8* methylation is associated with higher free T4, which, in turn, is associated with lower BMD.

The associations between *PAX8* methylation, thyroid phenotype and BMI, fat, and bone measures could reflect an adaptive process. For example, if higher PAX8 methylation [associated with a negative maternal energy balance in the Gambian rainy season (*34*)] is associated with a reduced level of thinness (as shown by a higher BMI in the high methylation group), then this could be an example of the organism developing greater fat stores in response to a predicted nutritionally adverse postnatal environment.

We found an inverse relationship between *PAX8* methylation and *PAX8-AS1* expression (measured by RSEM) in normal thyroid biopsies using data from the TCGA. However, we obtained contradictory results when *PAX-AS1* expression was measured using the FPKM metric (both expression measures are provided in the TCGA dataset). In general, these metrics are well correlated except when the exons are short (as for *PAX8-AS1*). There may be difficulty in accurately assessing the expression with short transcripts due to limitations with the sequencing technology and normalization methods (*58*); therefore, we present both metrics for consideration. DNA methylation in gene promoters is generally associated with condensed heterochromatin and reduced gene expression (*22*), while methylation within genes, downstream from the transcription start site (TSS), does not have as clear a correlation with gene expression (*59*). Our region of interest is intragenic to *PAX8* but is located within a promoter region of the antisense long noncoding RNA (lncRNA) *PAX8-AS1* (also referred to as *LOC654433*). At loci such as this, it is possible for complex regulatory interactions to exist between DNA methylation, coding gene expression, and noncoding RNA expression (*59*). A study in fibroblasts demonstrated that the relationship between methylation and gene expression at *PAX8* is region specific, with a positive correlation between methylation and expression reported at CpGs close to the *PAX8* TSS and a strong negative correlation reported at intragenic CpGs located near the *PAX8-AS1* TSS, including one of the CpGs in our region of interest (*60*). Furthermore, this study demonstrated a positive correlation between *PAX8* and *PAX8-AS1* gene expression and postulated a potential chromatin-activation linked role of *PAX8-AS1* in regulating *PAX8* expression. Previous studies have demonstrated that polymorphisms in *PAX8-AS1* are potentially expression quantitative trait loci for *PAX8* (*61*), and therefore, it appears that our region could be a putative intragenic regulatory region. The specific biological consequence of the *PAX8-AS1* lncRNA is still yet to be fully elucidated, but variants in *PAX8-AS1* are associated with cancer risk (*62*).

A previous study identified moderate iodine deficiency in Gambian children aged 8 to 12 years (*63*), and mothers recruited for the ENID trial whose offspring were followed up for this study were found to have moderate iodine deficiency (*64*). We therefore attempted to account for this in our analysis as iodine levels can influence thyroid volume and function. We found no difference between the *PAX8* methylation groups when a Tg cutoff was used to define iodine insufficiency, but we did find a significant difference between the groups when using UIC and therefore adjusted for this in the analysis. We recognize that a measure of urinary iodine can be a useful tool to understand iodine insufficiency in a population but may be less useful for characterizing an individual’s iodine status (*65*). However, we note that iodine levels were all taken at the same time of year, all in the morning from fasted individuals and where the local diet day to day is relatively consistent, suggesting that UIC may be informative. It is possible that differential *PAX8* methylation could be contributing to the observed differences in urinary iodine between the two groups possibly by influencing expression of the sodium-iodide symporter.

Prior evidence that *PAX8* methylation is a putative ME, with systemic methylation established in the early embryo, supports the notion that interindividual variation in *PAX8* methylation or related epigenetic marks may drive the phenotypic differences observed in this study. This highlights the potential benefits of studying links between regions of systemic interindividual variation and risk of disease (*66*). In this study, we cannot rule out the possibility of a reverse causation effect of thyroid function measures on methylation or other related epigenetic factors at 2 years of age. Elucidation of causal pathways linking environmental exposure, methylation and other epigenetic factors, gene expression, and postnatal phenotype will require mechanistic investigations in cell or animal models. For example, a mouse model has demonstrated an association between high estrogen exposure in early pregnancy and higher free T4, *PAX8* promoter hypomethylation, and increased PAX8 expression in offspring (*67*). However, we note that our region of interest is at least partially absent from several species commonly used as models of development including mouse, zebrafish, and *Xenopus tropicalis*. Furthermore, the *PAX8-AS1* lncRNA present in humans does not appear to exist in other species. Its putative role in regulating *PAX8* expression and thyroid development may therefore only exist in humans.

In summary, we have demonstrated that individual variation in DNA methylation at a region of the *PAX8* gene sensitive to periconceptional nutrition is significantly associated with total thyroid volume and free T4 levels in Gambian children. Our work has potential implications for understanding the fetal origins of health and disease and may contribute to our understanding of the epigenetic drivers of thyroid development and function.

## MATERIALS AND METHODS

### Experimental design

We used a “recall-by-epigenotype” design to examine links between *PAX8* DNA methylation measured at 2 years of age and thyroid gland function and development in the same Gambian children aged 5 to 8 years. We used an existing longitudinal cohort of Gambian children [children from the ENID (*37*) study now aged between 5 and 8 years, *n* = 493] to recruit the top (high) and bottom (low) quantiles for DNA methylation at a region of the PAX8 gene previously identified as sensitive to the periconceptional environment from banked DNA at 2 years of age. Participants were assessed for thyroid volume, function (free T3, free T4, TSH, and Tg), UIC, and body composition and bone measures by whole-body DXA scan. Figure 1 provides an overview of the study, and further details are provided below.

### ENID trial and cohort

The ENID trial (ISRCTN49285450) (*37*) was a combined trial of pregnancy and infancy nutritional supplementation conducted in West Kiang, a rural region of The Gambia, recruiting pregnant mothers between January 2010 and February 2014. Briefly, women were recruited in early pregnancy (10 to 20 weeks) and randomized to receive either (i) iron-folate (standard care); (ii) MMN; (iii) energy, protein, and lipid with iron-folate; or (iv) energy, protein, and lipid with MMN supplements for the remainder of their pregnancy. There were no differences in maternal (BMI, age, and parity) or infant characteristics (birthweight, birth length, sex, or gestational age) across the study arms (*68*). From 6 to 18 months of age, infants were further randomized to a lipid-based nutritional supplement, with or without additional MMN. A total of 875 women were randomized in pregnancy to one of the four study arms and 686 participants completed follow-up to 2 years of age (*69*). Routine blood samples were collected from mothers and infants at various time points including a peripheral blood cell DNA samples stored at age 2 years. At the time of this current study, children from the ENID study, now aged between 5 and 8 years of age (*n* = 493), were being followed up monthly in a separate longitudinal observational study (see Fig. 1).

### PAX8 methylation measurement at 2 years of age

Children from the original ENID trial had DNA isolated from whole blood at age 2 years. DNA was enriched for a panel of candidate regions and bisulfite-converted using a custom Agilent SureSelect Methyl-Seq targeted capture system on a subset of these children with sufficient DNA available for processing (*n* = 521) (*70*). Target-enriched DNA, including the *PAX8* region of interest, was sequenced using the Illumina NovaSeq platform at the Human Genome Sequencing Center, Baylor College of Medicine, Houston, Texas, USA. Reads were mapped to the human genome (hg38) using Bismark v0.20.0 (*71*) with default options, which was also used to extract methylation values after mapping. Methylation calls from opposite strands of the same CpG site were combined. Within each individual, CpG sites were considered “covered” if they had a read depth of at least 20×; uncovered sites were excluded from analyses.

### Selection of PAX8 region of interest

We selected a subset of CpGs in the *PAX8* region for which there was independent evidence of systemic interindividual variation and sensitivity to early environment from previous studies (*23*, *25*, *27*). From these, four CpGs of interest, with coverage in a large number of samples and strong correlation with nearby CpGs, were chosen (see fig. S10): chr2:113,235,186; chr2:113,235,228; chr2:113,235,251; and chr2:113,235,267. All of these CpGs lie in intron 9 of *PAX8* and in a promoter region of antisense *PAX8-AS1* (see Fig. 2; all genomic coordinates hg38).

### Participant selection

From the 493 children being followed up in the ENID cohort at age 5 to 8 years (see Fig. 1), a “recall by epigenotype” design was used whereby participants were selected by methylation level at CpG chr2:113,235,228. This CpG was chosen due to it having the best overall correlations with nearby CpG methylation levels (see fig. S10). Individuals with at least one other informative CpG among the four CpGs of interest and without large differences (≥0.2) in quantile among the CpGs of interest (*n* = 217) were then placed into high or low groups based on the chr2:113,235,228 methylation level (see Fig. 3). In total, 125 participants (low *PAX8* methylation group *n* = 64, high *PAX8* methylation group *n* = 61) were identified for potential recruitment, with 118 participants (94%) consenting to participate in the study (*n* = 7 declined to participate).

### Thyroid volume assessment

Thyroid ultrasound was conducted by T.C. who was blinded to the participant’s *PAX8* methylation group. The length (*l*), width (*w*), and depth (*d*) of each thyroid lobe (in centimeters) were measured on transverse and longitudinal scans using Sonosite MicroMaxx (10-Hz probe). All measurements were made in triplicate, and the mean was used for analysis. The volume (Vol) of each lobe (in milliliters) was estimated by the modified formula for an ellipsoid; Vol(ml) = (0.479 × *d* × *w* × *l*) (*72*), and the total thyroid volume was calculated as the sum of the volumes of both lobes.

### Mid-childhood blood collection and biochemical assessments

Recruited children had a morning (between 8 a.m. and 10.30 a.m.) venous blood sample taken into a serum sample collection tube in April 2019. Aliquoted serum was frozen at −70°C. The cellular fraction was discarded. During the same study visit as the blood sample and thyroid ultrasound, a fasted morning urine sample was collected in iodine-free tubes and stored at −20°C. The urine and serum samples were shipped to the University Hospital of Wales, UK for thyroid hormone and urinary iodine measurements. Serum TSH, free T3, and free T4 were measured by the automated ALINITY System (Abott Laboratories, USA), and Tg was measured by the Beckman Access DxI. Urinary iodine and urinary creatinine were measured by inductively coupled plasma mass spectrometry. Iodine sufficiency was defined as a Tg < 40 μg/liter and/or a UIC > 100 μg/liter (*63*).

### Other phenotypic measures

Standing height was calculated as the mean of measures taken in triplicate to the nearest millimeter using a portable stadiometer (Seca 213). Weight was similarly calculated from measures in triplicate to the nearest 0.1 kg using electronic scales (Seca 803), with participants clothed, but with shoes and coat removed. BMI was calculated as weight (kilograms) divided by height^2^ (m^2^). HAZ, WAZ, and BMI SD score for each participant were calculated using World Health Organization reference ranges (*73*). A whole-body DXA scan was performed using the GE-Lunar Prodigy scanner (GE Medical, Waltham, MA; software version 13.60.033) on 113 children (5 did not attend for DXA scan). Bone-related outputs included areal BMD (g/cm^2^), bone mineral content (BMC, g), and bone area (BA, cm^2^). For children, it is recommended to use TBLH for bone-related measurements (*74*). The analyzed outcome measure for bone was TBLH BMD and was calculated as TBLH BMD (g/cm^3^) = TBLH BMC (g)/ TBLH BA (cm^2^). The analyzed outcome measure for body fat was FMI, which was calculated as fat mass (kg) divided by height^2^ (m^2^). The analyzed outcome measure for lean mass was LMI, which was calculated as lean mass (kg) divided by height^2^ (m^2^).

### Maternal biomarker data

Women recruited to the ENID trial provided a 10-ml venous blood sample at the time of enrolment in early pregnancy (<20 weeks of gestation). Plasma was stored at −70°C. A subset of 350 women were previously selected for biomarker analysis on the stored samples; *n* = 303 women with maternal biomarker data had *PAX8* methylation data available for their offspring. These women were selected to give an even distribution by month of enrolment and to provide data from the earliest gestational age, i.e., sample collection nearest to time of conception (median 12.1 weeks, IQR 3.5). In our analysis, we examined a core set of 10 nutritional biomarkers involved in one-carbon metabolism [homocysteine, methionine, cysteine, choline, betaine, dimethylglycine (DMG), vitamin B12, folate, PLP, and vitamin B2 (riboflavin)]. We also considered an extended panel of biomarkers to capture other metabolic pathways potentially influencing one-carbon metabolism [alpha-1-acid glycoprotein (AGP), aspartate, threonine, serine, glutamate, glycine, alanine, valine, isoleucine, leucine, tyrosine, phenylalanine, lysine, histidine, arginine, proline, uridine, and uracil]. Biomarkers were analyzed at the BC Children’s Hospital, Canada, using liquid chromatography–tandem mass spectrometry (choline, betaine, DMG, homocysteine, cysteine, methionine, PLP, riboflavin, uracil, and uridine), Abbott AxSYM autoanalyzer (folate and vitamin B12), and Hitachi L-8900 amino acid analyzer (additional amino acids: serine, glycine, alanine, arginine, aspartic acid, glutamic acid, histidine, isoleucine, leucine, lysine, phenylalanine, proline, threonine, tryptophan, tyrosine, and valine). The inflammatory marker AGP was measured using the Cobas Integra 400 plus autoanalyzer at the MRC Unit The Gambia, Keneba field station.

### Stability of PAX8 methylation from mid-childhood to after puberty/adolescence

Stability of the four selected *PAX8* CpGs with age was assessed by analyzing blood samples from 49 Gambians with samples collected in mid-childhood (7 years old) and adolescence (17 years old) (*25*) by pyrosequencing (see Supplementary Methods).

### PAX8 gene expression and DNA methylation in blood and thyroid tissues

We accessed data from TCGA, an open access resource providing genomic data for more than 20,000 cancer and noncancerous matched samples obtained from biopsy from live individuals. We downloaded *PAX8* gene expression data on 50 individuals with noncancerous thyroid samples, reported as both FPKM upper quartile (from GDC v18.0 PANCAN HTSeq hosted by Xena, https://gdc.xenahubs.net) and RSEM (data from TCGA Wanderer, http://maplab.imppc.org/wanderer/) from Illumina HiSeq RNA-seq data. TCGA methylation data for the same samples measured on the Illumina HumanMethylation450 array were available for two of our CpGs of interest (chr2:113,235,186 and 113,235,267) from TCGA Wanderer.

A comparison of *PAX8* methylation levels in blood and thyroid tissue was carried out through quantitative analysis of the four *PAX8* CpGs of interest by bisulfite pyrosequencing in paired thyroid tissue, and whole blood samples from 86 donors were obtained from the GTEx postmortem tissue database (*38*) (see Supplementary Methods for sample details).

### Genotyping from methyl-seq data

We used the targeted methyl sequencing (methyl-seq) data to call individual genotypes within the *PAX8* gene. A modified version of BS-Snper (*75*, *76*) was used to call SNP genotypes from the bisulfite-converted reads. Genotypes with a nonreference allele frequency (AF) of ≥0.05 and with a call rate (proportion of individuals with called genotypes) of 20% or higher were considered for the analysis (*n* = 14 SNPs). Of these, SNP rs10193733 (chr2:113,235,047 T > C; MAF, 0.25) was the closest to the *PAX8* variably methylated region analyzed here (see Fig. 2) and had a high call rate in the methyl-seq dataset (>99% of available samples with methylation data; *n* = 523), enabling analysis of interactions between methylation, genotype, and thyroid phenotypes. Furthermore, we verified that this SNP is in LD with several other SNPs on either side of the variably methylated region using Gambian reference data from the Gambian Genome Variation Project (GGVP) (*77*) and 1000 Genomes Project, Phase3 [Gambia West Division (GWD)] (*78*) and confirmed this in our study population (see fig. S11). This SNP and the nearby SNPs in LD all had similar AFs to the GWD reference population from GGVP, demonstrating the reliability of the methyl-seq–derived genotypes (with the exception of rs7576384 chr2:113235808 C > G, which had a much lower AF than expected, likely due to the loss of informative reads after bisulfite conversion). A 2–base pair (bp) indel polymorphism not detectable by the genotype caller, rs35724515 (chr2:113235224 CCC > C), is located within our region of interest and is in high LD with rs10193733 (1000 Genomes GWD LD *r*^2^ = 0.976).

### Statistical analysis

#### Baseline characteristics and crude between-group comparisons

Differences between high and low *PAX8* methylation groups for the categorical variables of sex, pregnancy and infant supplementation, and iodine sufficiency (yes/no) were assessed using Pearson chi-square tests. Birth weight, WAZ, BMI *z* score, HAZ, total thyroid volume, free T4, and free T3 were normally distributed, and group differences were assessed using Student’s *t* test with mean and SD reported. Age, UIC, urinary iodine:creatinine ratio, TSH, and Tg were not normally distributed, and group differences were assessed by Mann-Whitney *U* tests with median and IQR reported.

#### Multiple linear regression models

Unless otherwise indicated, all outcome variables were normally distributed.

##### Thyroid volume

Thyroid volume as the dependent variable was regressed against *PAX8* methylation group and adjusted for sex, age, BMI, and UIC. BMI was included to ensure an adjustment for body size as (i) there was a significant difference between the two groups at recruitment and (ii) BMI also provided the best model fit (indicated by lowest Akaike information criterion) compared to other measures of body size (e.g., WAZ, HAZ, and body surface area). UIC was included as there was a significant difference between the groups at recruitment and iodine deficiency can be associated with goiter/thyroid size.

##### Measures of thyroid function

Measures of thyroid function (free T4, free T3, TSH, and Tg) were regressed against *PAX8* methylation group and adjusted for sex, age, and UIC. TSH and Tg were normally distributed after log transformation before regression analysis. Iodine deficiency can be associated with hypothyroidism and an increase in Tg, and therefore, UIC was included in all models. Correction for multiple tests was not made as the markers of thyroid function are not independent.

##### DXA-derived measures

FMI was normally distributed after log transformation. FMI was regressed against free T4 and adjusted for age, sex, and weight (to adjust for lean mass). LMI was regressed against free T4, with LMI adjusted for age, sex, and weight (to adjust for fat mass). TBLH BMD was normally distributed after log transformation and regressed against free T4, adjusted for age, sex, weight, and height.

#### Predictors of PAX8 methylation analysis

In analyses where *PAX8* methylation was considered as an outcome, 2-year methylation measurements were taken from the wider ENID cohort (i.e., not just the high and low *PAX8* methylation groups; *n* = 303 to 521 depending on the predictor considered) and treated as a continuous variable. Methylation across the four CpGs of interest was highly correlated (Pearson *R* between 0.871 and 0.952; fig. S10). A univariate composite measure was therefore used in all regression models, calculated as the mean *z* score (over all CpG sites) of the logit-transformed methylation level at each CpG site (referred to as *PAX8* mean logit methylation *z* score). Methylation levels of 1.0 were reduced to 0.99 to prevent infinite values after logit transformation.

All maternal biomarkers were preadjusted for gestational age, maternal BMI, maternal age, and inflammation (AGP) and then back-extrapolated to date of conception using previously described methods (*29*). Those biomarkers not normally distributed were log-transformed, and all biomarkers were scaled and centered to enable comparison of standardized coefficients. Multiple linear regression models with *PAX8* mean logit methylation *z* score as the dependent variable were fitted individually with each biomarker as a predictor and adjusted for sex.

For other predictors, *PAX8* mean logit methylation *z* score was regressed against maternal BMI, infant BMI *z* score, infant WAZ, infant sex, and SoC in separate models. SoC was defined as “rainy” (January to June) and “dry” (July to December) as previously described (*79*) with the conception date calculated from a gestational age estimation obtained from antenatal ultrasound at ENID trial recruitment.

All model covariates were assessed for multicollinearity, and standard tests were performed to ensure that linear modeling assumptions were met. Where reported, coefficients (β) associated with log-transformed dependent variables were back transformed using (exp(β) − 1) × 100 to represent percentage change in dependent variable per unit increase in the corresponding predictor.

### Causal mediation analysis

We performed a causal mediation analysis to test the hypothesis that the observed effect of *PAX8* methylation group on free T4 is mediated by its effect on thyroid volume. We used the Mediation package (v4.5.0) in R, with confidence intervals for direct and indirect effects calculated using a nonparametric bootstrap with 10,000 simulations. All statistical analyses were performed using R version 3.6.2 (*80*).

### Analyses by genotype

Genetic analyses focused on a single SNP (rs10193733) that tagged an LD block proximal to our *PAX8* region of interest (see above for justification for choosing this SNP). We performed two sets of analyses stratified by rs10193733 genotype. First, Student’s *t* tests were used to compare the *PAX8* mean logit methylation *z* score between SoC (rainy versus dry) separately for individuals with each genotype. Second, we fitted multiple linear regression models with total thyroid volume and free T4 as dependent variables adjusted for relevant covariables, again stratified by rs10193733 genotype. We also assessed the influence of the rs10193733 “C” allele on free T4 and thyroid volume (outcome variables) in multiple regression models with allelic dosage (predictor) coded as C/C = 2, C/T = 1, T/T = 0.

### Ethics

Ethical approvals for the ENID trial and for this study were given by The Gambia Government/MRC Joint Ethics Committee (SCC1126v2, SCC1640, and L2015.51v2). Consent was gained by signature or thumb print from mothers for their own participation and that of their child. All data were anonymized before analysis.

## Notes

View/request a protocol for this paper from *Bio-protocol*.
