# Supplementary material for: DNA methylation at a nutritionally sensitive region of the PAX8 gene is associated with thyroid volume and function in Gambian children
Source: Sci Adv. 2021 Nov 5;7(45):eabj1561. doi: 10.1126/sciadv.abj1561 (PMC8570597; doi:10.1126/sciadv.abj1561)
Supplement: Supplementary file 1 — Supplementary Methods Tables S1 to S8 Figs. S1 to S11 [file sciadv.abj1561_sm.pdf]

Science Advances
AAAS

Supplementary Materials for

DNA methylation at a nutritionally sensitive region of the *PAX8* gene is associated with thyroid volume and function in Gambian children

Toby Candler, Noah Kessler, Chathura Gunasekara, Kate Ward, Philip James, Eleonora Laritsky, Maria Baker, Roger Dyer, Rajavel Elango, David Jeffries, Robert Waterland, Sophie Moore, Marian Ludgate, Andrew Prentice, Matt Silver*

*Corresponding author. Email: matt.silver@lshtm.ac.uk

Published 5 November 2021, *Sci. Adv.* **7**, eabj1561 (2021)
DOI: 10.1126/sciadv.abj1561

This PDF file includes:

Supplementary Methods
Tables S1 to S8
Figs. S1 to S11

Supplementary Tables

Study	Exposure	Timing of prenatal exposure	Offspring *PAX8m* methylation outcome	Age of offspring's *PAX8m* measurement	Methylation platform
Waterland et al, 2010(31)	Gambian Season of Conception	Periconceptional	*Rainy season conceptions associated with ↑ leucocyte *PAX8*m	Children (~9 years of age)	Pyrosequencing
Silver et al, 2015(25)	Gambian Season of Conception	Periconceptional	*Rainy season conceptions associated with ↑ leucocyte *PAX8*m	Infants (~3 months of age)	Illumina Infinium Human Methylation 450 array
Finer et al, 2016(27)	Famine Exposure in Bangladesh	At least 7 months of pregnancy	Gestational famine exposure associated with ↑ leucocyte *PAX8*m	Adults (27-32 years of age)	Illumina Infinium Human Methylation 450 array
Richmond et al, 2018(35)	Maternal folic-acid supplementation in pregnancy	Folic acid supplementation or placebo given between ~17 weeks GA (mean GA at study recruitment) to ~40 weeks GA	Maternal folic acid supplementation associated ↓salivary *PAX8*m	Adults (46-48 years of age)	Illumina Infinium Human Methylation 450 array
Saffari et al, 2020(36)	Micronutrient supplementation (UNIMMAP) in pregnancy	Preconception to positive pregnancy test	Micronutrient supplementation associated with ↑ leucocyte *PAX8*m	Children (~9 years of age)	Pyrosequencing

Supplementary Table 1. Summary of previous studies investigating associations between prenatal environmental or nutritional exposures and *PAX8* methylation. *Rainy season conceptions occur in the context of lower maternal calorific intake and altered concentrations of circulating C1 metabolites[34,29].
Key: PAX8m = *PAX8* gene methylation, C1 = one-carbon, GA=gestational age, UNIMMAP= United Nations International Multiple Micronutrient Antenatal Preparation

	Total Thyroid Volume (cm^3)
**Age (Years)**	0.38*** [0.07]
**Sex (Male)**	-0.32* [0.16]
**BMI Z score**	0.22* [0.09]
PAX8 **Methylation Category (Low)**	0.61*** [0.15]
**Urinary Iodine (µg/L)**	-0.0002* [0.0001]

Supplementary Table 2. Multiple linear regression models for predictors of total thyroid volume.

Coefficients from regression model are given with [standard error]. *p<0.05; **p<0.01; ***p<0.001.

Key: BMI = body mass index

Iodine Status				
	**All**	**High *PAX8* methylation**	**Low *PAX8* methylation**	**p-value**
**Median UIC [IQR] ug/L**	152 [141] n=116	128 [161] n=56	170 [140] n=60	**0.04**
**Median urinary iodine:creatinine ratio [IQR] nmol/mmol**	189 [113] n=116	190 [100.96] n=56	186.71 [153.79] n=60	0.65
**Iodine insufficient by Tg level**	9 [8.6%]	1 [2%]	8 [14.3%]	0.06
**Iodine sufficient by Tg level**	96 [91.4%]	48 [98%]	48 [85.7%]	
**Iodine insufficient by UIC**	31 [26.7%]	21 [37.5%]	10 [16.7%]	**0.02**
**Iodine sufficient by UIC**	85 [73.3%]	35 [62.5%]	50 [83.3%]	

Supplementary Table 3. Iodine status by *PAX8* methylation groups

Group differences in non-normally distributed variables (TSH, Tg, urinary iodine, urinary iodine:creatinin e ratio) were assessed by Mann-Whitney U test, categorical variables (iodine sufficiency categories) by ch i-squared test.

Key: Tg = thyroglobulin, UIC = Urinary Iodine concentration. Iodine insufficiency by Tg is defined as a Tg level >40ug/L. Iodine insufficiency by UIC is defined as a UIC <100ug/L.

	Free T4 (pmol/L)	Free T3 (pmol/L)	Log TSH (mU/L)	Log Tg (µg/L)
**Age (Years)**	-0.04 [0.12]	-0.19^{**} [0.06]	-0.02 [0.05]	-0.05 [0.06]
**Sex (Male)**	-0.73^{**} [0.25]	-0.08 [0.13]	-0.1^{*} [0.10]	-0.001 [0.12]
***PAX8* Methylation Category (Low)**	0.85^{***} [0.24]	-0.02 [0.13]	-0.11 [0.09]	0.02 [0.12]
**Urinary Iodine (µg/L)**	-0.0002 [0.0001]	0.00003 [0.0001]	0.0001 [0.00004]	-0.0001^{**} [0.0001]

Supplementary Table 4. Multiple linear regression models for predictors of free T4, free T3, TSH and Tg.

Coefficients from regression models are given with [standard error]. *p<0.05; **p<0.01; ***p<0.001.

Key: Free T4 = free thyroxine, free T3 = free tri-iodothyronine, TSH = thyroid stimulating hormone, Tg = thyroglobulin.

	Log FMI (fat mass in kg)/m²)
**Age (Years)**	-0.14*** [0.04]
**Sex (Male)**	-0.53*** [0.06]
**Weight (Kg)**	0.06*** [0.01]
**Free T4 (pmol/L)**	-0.04* [0.02]

Supplementary Table 5. Multiple linear regression models for predictors of fat measures as assessed by DXA.

Coefficients from regression model are given with [standard error]. *p<0.05; **p<0.01; ***p<0.001.

Key: FMI = fat mass index

	LMI (Lean Mass (Kg)/m^2)
**Age (Years)**	-0.21* [0.08]
**Sex (Male)**	0.82*** [0.13]
**Weight (Kg)**	0.19*** [0.03]
**Free T4 (pmol/L)**	-0.02 [0.05]

Supplementary Table 6. Multiple linear regression models for predictors of lean measures as assessed by DXA.

Coefficients from regression model are given with [standard error]. *p<0.05; **p<0.01; ***p<0.001.

Key: LMI = lean mass index

	Log TBLH BMD (g/cm^2)
**Sex (Male)**	-0.01 [0.01]
**Age (Years)**	0.02 [0.01]
**Height (cm)**	0.003 [0.002]
**Weight (Kg)**	0.01* [0.003]
**Free T4 (pmol/L)**	-0.008* [0.004]

Supplementary Table 7. Multiple linear regression models for predictors of BMD as assessed by DXA.

Coefficients from regression model are given with [standard error]. *p<0.05; **p<0.01; ***p<0.001.

Key: TBLH = total body less head, BMD = bone mineral density.

Covariate	Standardised Coefficient	Standard Error	t-value	p-value
**AGP (g/L)**	0.02	0.05	0.42	0.63
**Aspartate (µmol/L)**	0.05	0.06	0.91	0.37
**Threonine (µmol/L)**	-0.05	0.06	-0.84	0.40
**Serine (µmol/L)**	-0.08	0.06	-1.40	0.16
**Glutamate (µmol/L)**	0.08	0.06	1.38	0.17
**Glycine (µmol/L)**	-0.05	0.06	-0.93	0.35
**Alanine (µmol/L)**	0.05	0.06	0.79	0.43
**Valine (µmol/L)**	-0.13	0.05	-2.37	**0.02**
**Isoleucine (µmol/L)**	0.01	0.06	-0.26	0.80
**Leucine (µmol/L)**	0.02	0.06	0.32	0.75
**Tyrosine (µmol/L)**	0.04	0.06	0.79	0.43
**Phenylalanine (µmol/L)**	0.10	0.06	1.78	0.08
**Lysine (µmol/L)**	-0.01	0.06	-0.20	0.85
**Histidine (µmol/L)**	0.03	0.06	0.55	0.58
**Arginine (µmol/L)**	0.16	0.06	2.77	**0.006**
**Proline (µmol/L)**	0.04	0.06	0.72	0.47
**Uridine (µmol/L)**	-0.04	0.06	-0.78	0.43
**Uracil (nmol/L)**	0.21	0.05	3.87	**0.0001**

Supplementary Table 8. Linear regression models for additional nutritional predictors of *PAX8* Methylation.

Dependent Variable = *PAX8* Mean Logit Methylation z-score. All biomarkers measured in maternal plasma and back-extrapolated to conception and adjusted for sex.

Key: AGP = Alpha-1-acid glycoprotein.

Supplementary Figures

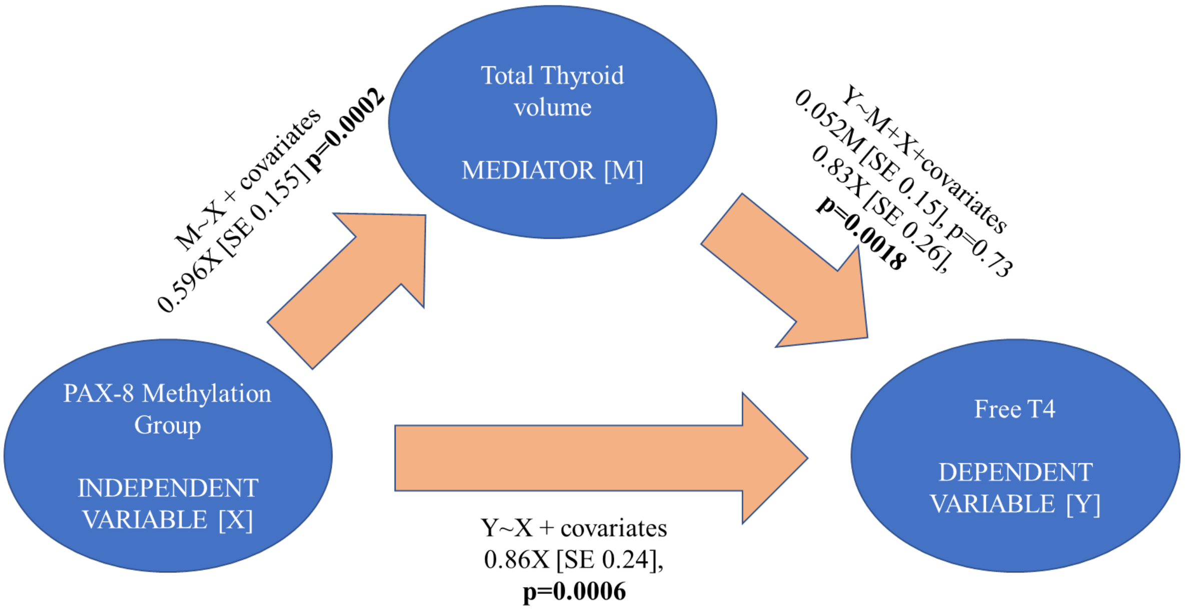

	Estimate	95% CI Lower	95% CI Upper	p-value
**Average causal mediation effect**	0.03	-0.15	0.22	0.74
**Average direct effect**	0.83	0.34	1.34	**0.001**
**Total effect**	0.86	0.39	1.35	**0.0002**
**Proportion mediated (%)**	0.035	-0.22	0.30	0.74

Supplementary Figure 1. Causal Mediation Analysis of the effect of *PAX8* methylation (X) on free T4 (Y), mediated by thyroid volume (M).

In each case only coefficients [SE] for relevant predictors (X or M) are given.

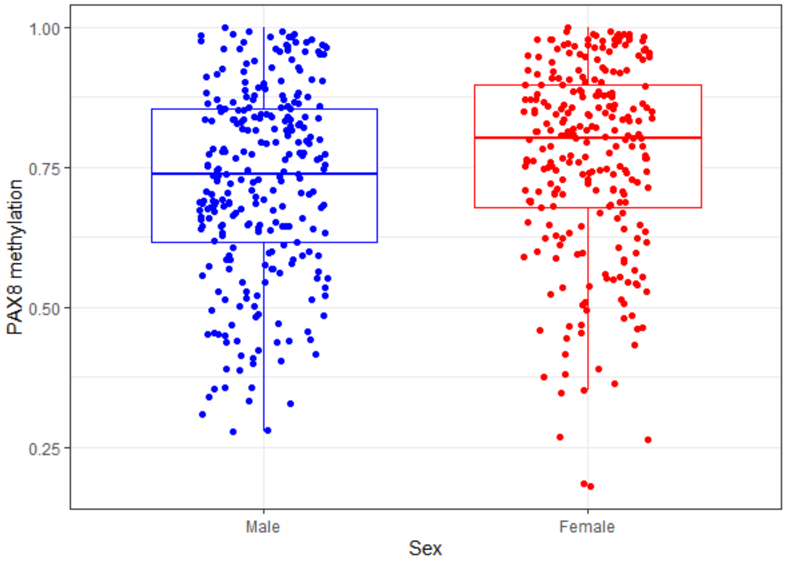

Supplementary Figure 2. Boxplot of mean *PAX8* methylation by Sex.

Mean methylation is calculated across the 4 CpGs in the *PAX8* region of interest. N=521 children.

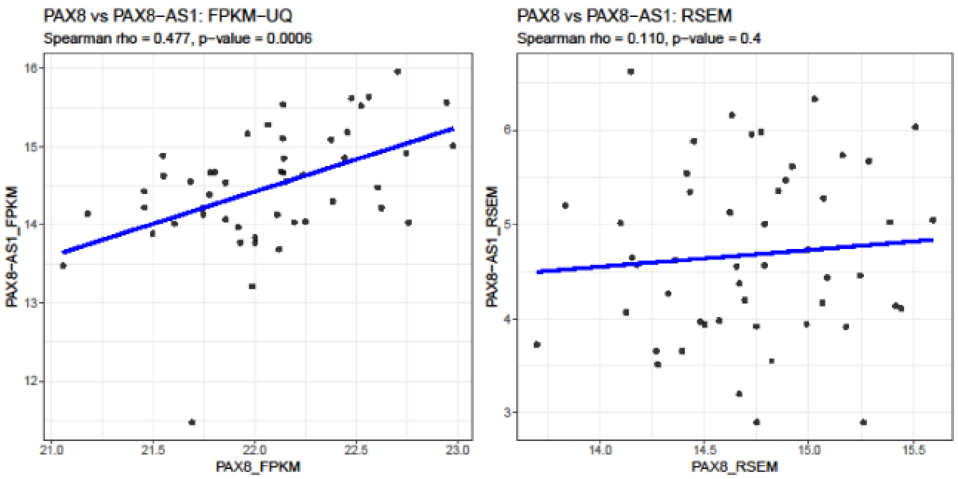

Supplementary Figure 3. Relationship between *PAX8* and *PAX-AS1* expression.

Correlation between *PAX8* and *PAX8-AS1* expression in normal thyroid tissue (n=50) using data downloaded from The Cancer Genome Atlas (TCGA). Spearman correlation coefficients and p-values are shown in the graphs.

Key: FPKM = fragments per kilobase per million mapped reads, RSEM= RNAseq by Expectation-Maximization, *PAX AS1 = PAX8 antisense*.

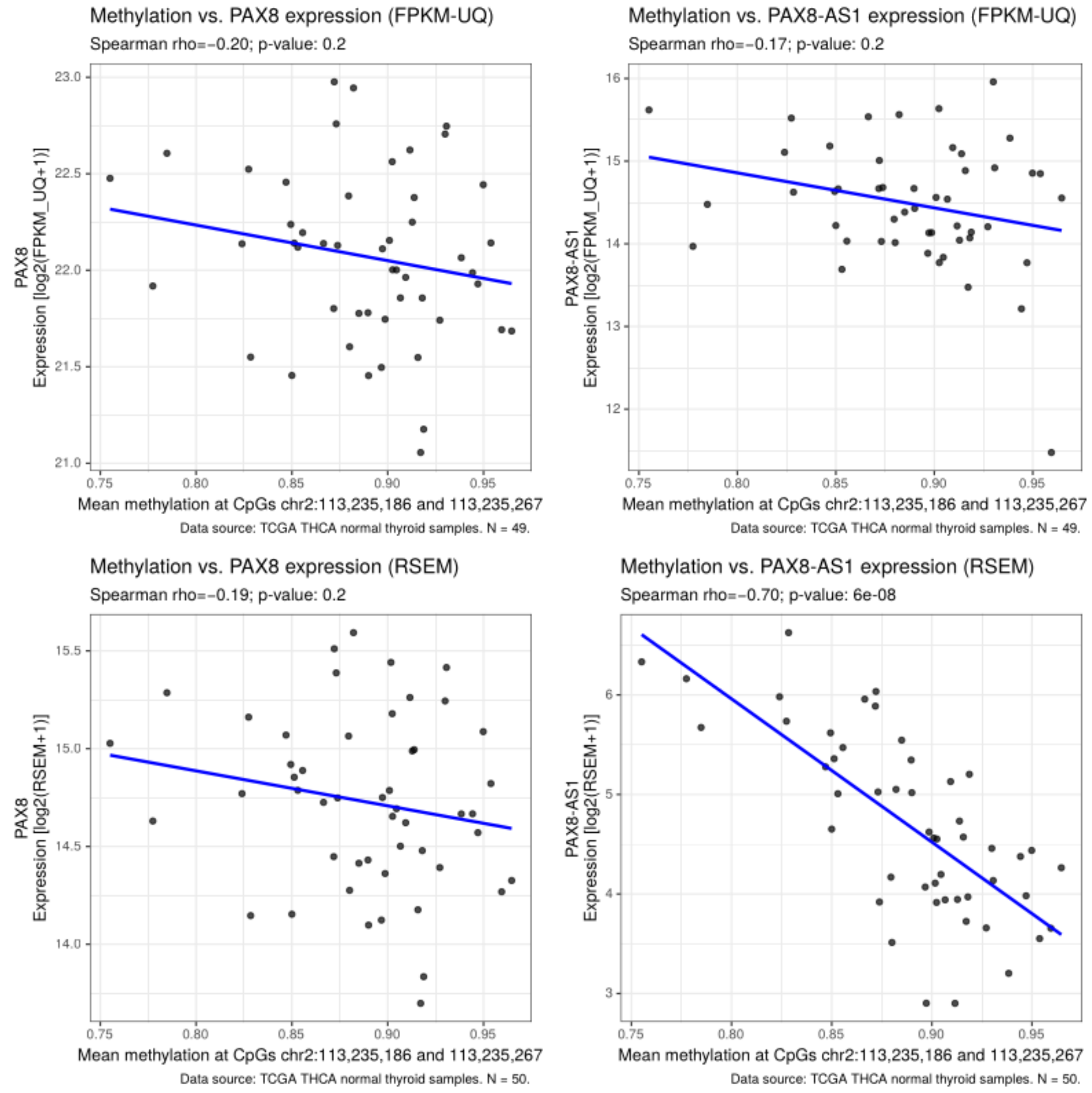

Supplementary Figure 4. Relationship between methylation and expression for *PAX8* and *PAX8-AS1* in TCGA thyroid samples.

Top: Correlation between *PAX8* and *PAX8-AS1* gene expression (using FPKM-UQ method) and mean methylation from normal thyroid tissue samples (n=49).

Bottom: Correlation between *PAX8* and *PAX8-AS1* gene expression (using RSEM method) and mean methylation from normal thyroid tissue samples (n=50). Data is downloaded from The Cancer Genome Atlas (TCGA), and covers 2 CpGs in our region of interest. Spearman correlation coefficients and p-values are shown in the graphs. Plots show mean methylation across the 2 CpGs.

Key: FPKM-UQ = fragments per kilobase per million mapped reads – upper quartile; RSEM = RNAseq by Expectation-Maximization.

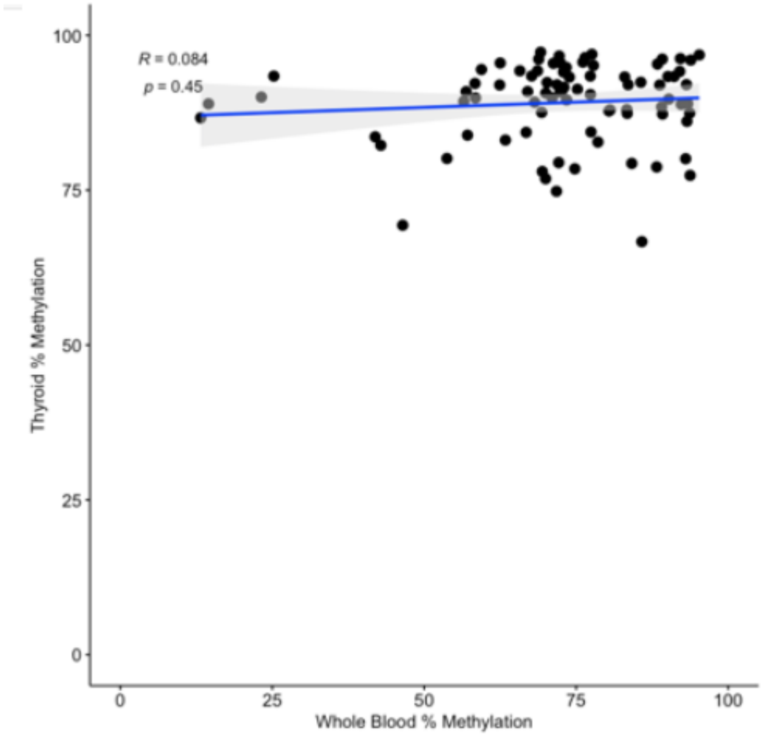

Supplementary Figure 5. Relationship between *PAX8* methylation in adult blood and thyroid samples from GTEx.
Correlation between mean whole blood and thyroid tissue methylation at 4 CpGs in the *PAX8* region of 86 paired samples from the GTEx biobank. Spearman correlation and p-value is shown.

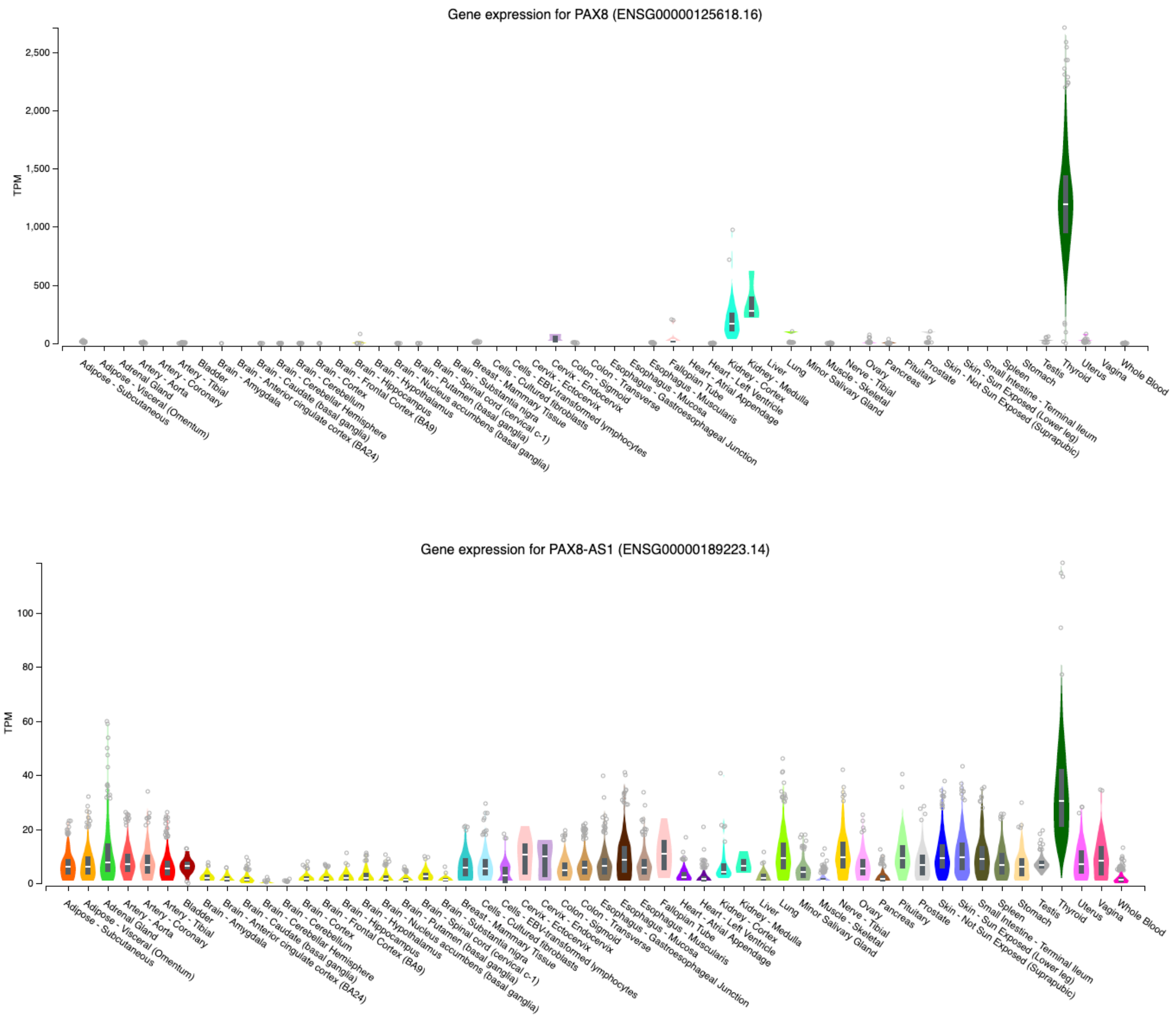

Supplementary Figure 6. Expression of PAX8 and PAX8-AS1 by tissue type.

PAX8 (top) and PAX8-AS1 (bottom) gene expression plots from the GTEx (Genotype-Tissue Expression) Project portal (https://www.gtexportal.org/).

Key: Transcripts per million = TPM, *PAX8-AS1* = *PAX8* antisense.

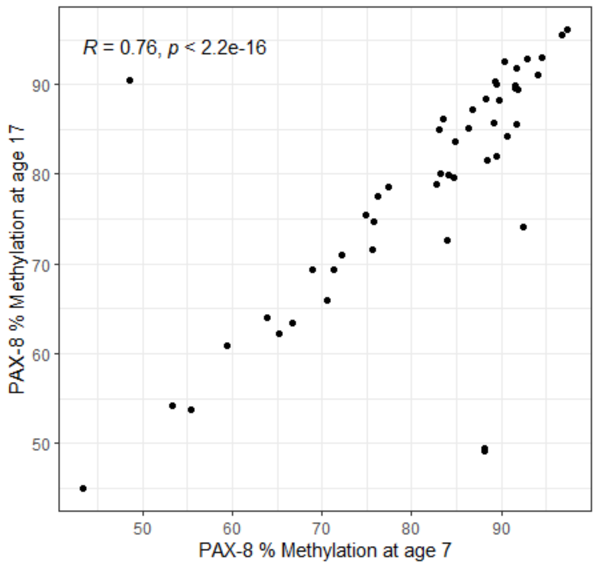

Supplementary Figure 7. Scatterplot of mean *PAX8* methylation measured in peripheral blood DNA from Gambian children in mid-childhood (aged 7 years) and in young adulthood (aged 17 years).

Spearman correlation coefficient and p-value are shown. N=49.

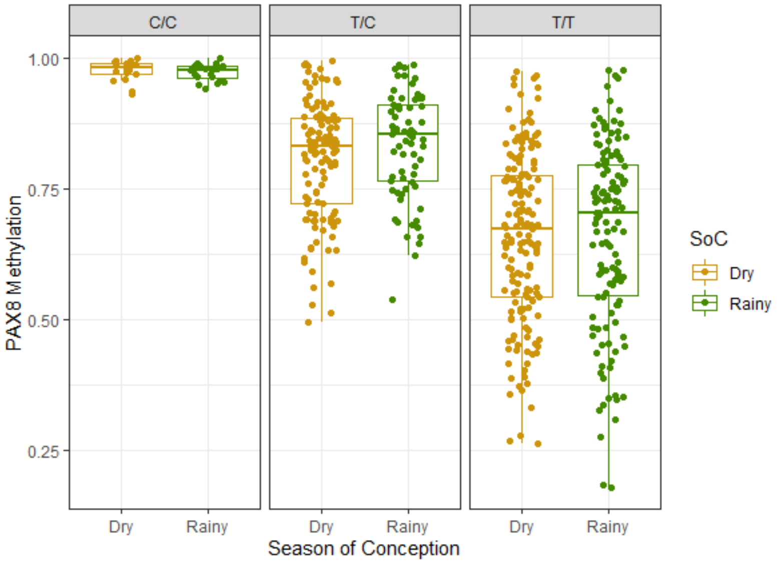

Supplementary Figure 8. Effect of season of conception on mean methylation at the *PAX8* region of interest, stratified by rs10193733 genotype.

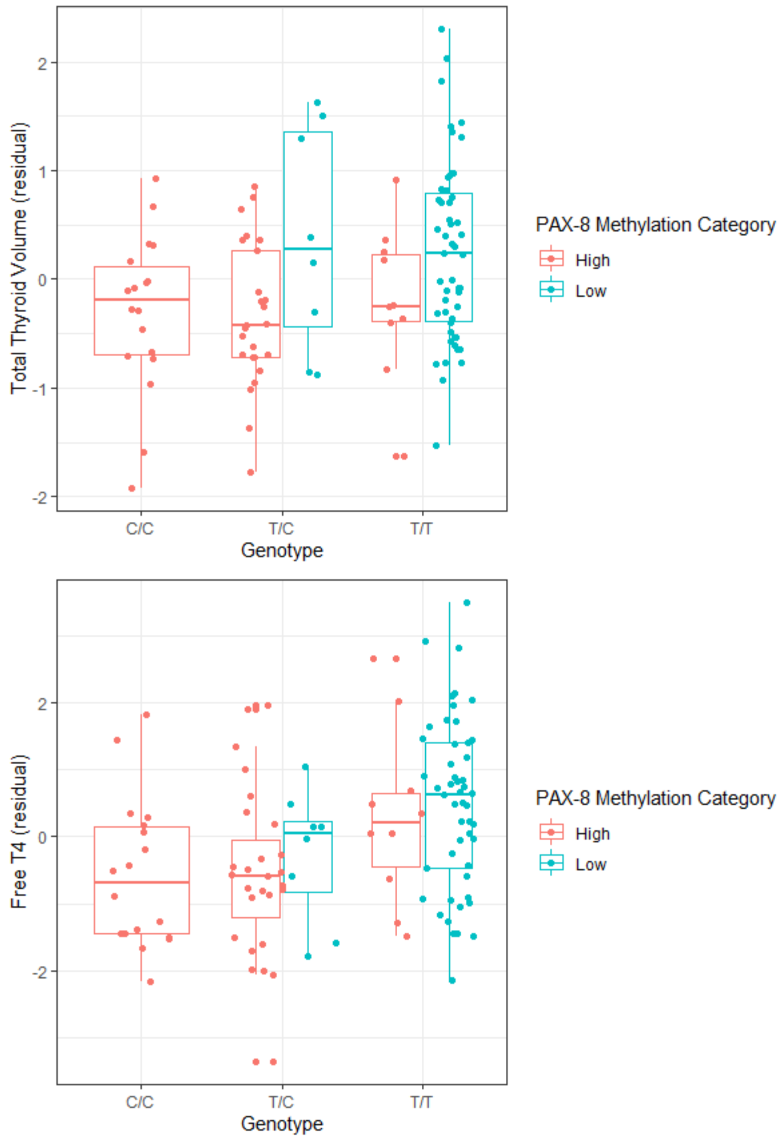

Supplementary Figure 9. Relationship between *PAX8* methylation group, total thyroid volume and free T4, stratified by rs10193733 genotype.

Top: Total thyroid volume is expressed as a residual adjusted for age, sex, BMI z-score and urinary iodine concentration (UIC).

Bottom: Free T4 is expressed as a residual adjusted for age, sex, and UIC.

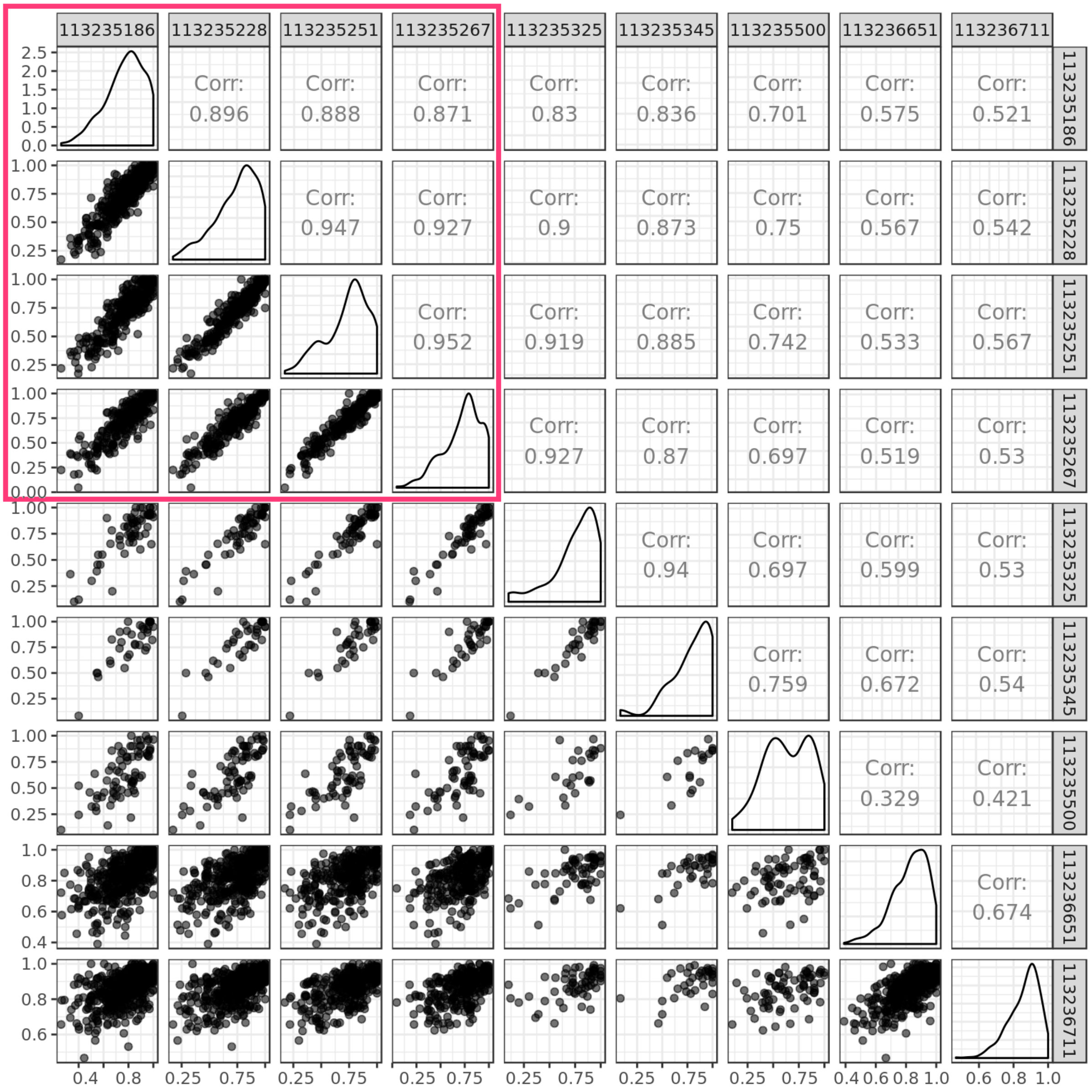

Supplementary Figure 10. CpG – CpG correlations across the *PAX8* region.

Genomic regions are mapped to hg38 on chromosome 2. Waterland et al, 2010(31) region: 113,235,685 - 113,235,814 and 113,235,289 - 113,235,459. Finer et al, 2016(27) and Silver et al, 2015(25) region: 113,235,185 - 113,235,736. Kessler et al, 2018(23) region: 113,235,117 - 113,236, 423. The final region of interest selected for analysis is marked by the red box.

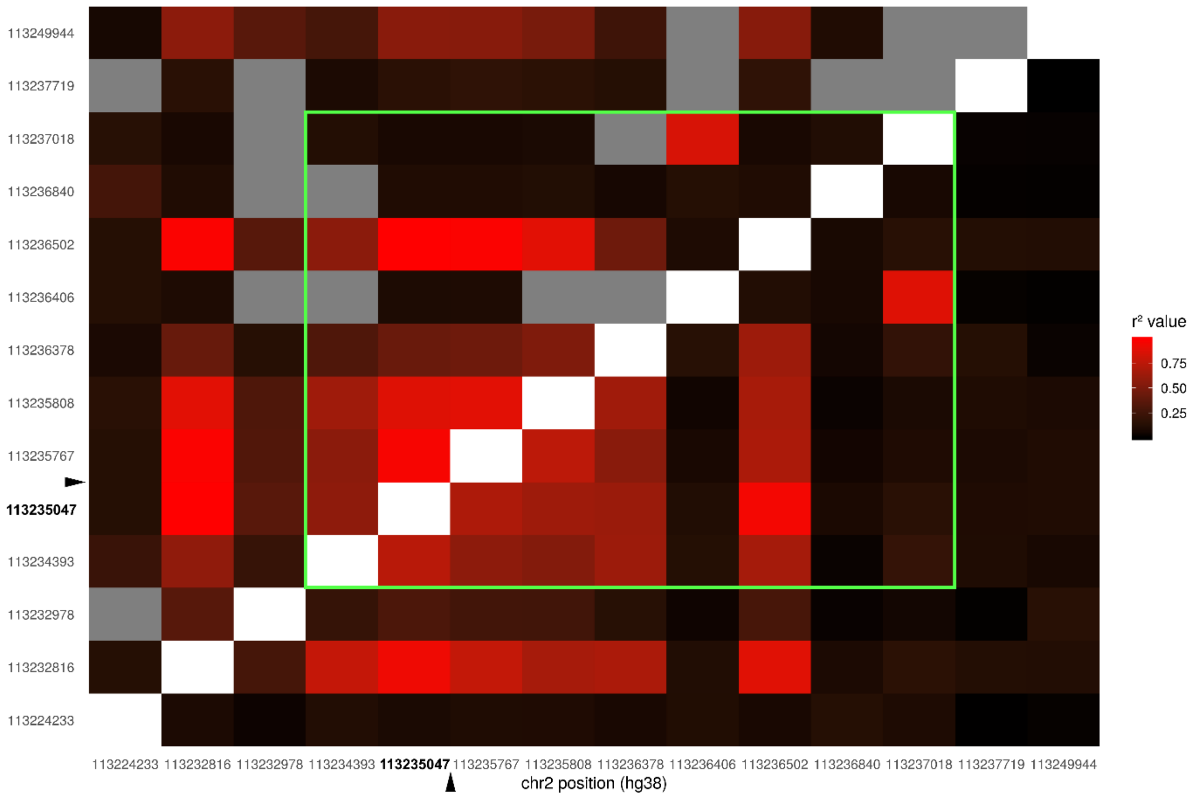

Supplementary Figure 11. Pairwise linkage disequilibrium (LD) of 14 SNPs located near the *PAX8* gene called from methyl-seq data.

Genomic regions are mapped to hg38 on chromosome 2. LD r^2 from reference populations (GGVP or 1000 Genomes where missing; above and left of diagonal); Pearson R^2 reported for ENID methyl-seq data (bottom and left of diagonal). Grey boxes indicate LD values missing from both reference datasets. Green box encloses SNPs denoted in Figure 2. The SNP (rs10193733; chr2:113235047) used in genotype analyses is indicated in bold, the approximate location of the PAX8 region of interest analysed in this study is indicated by the black triangles.

Supplementary Methods

Pyrosequencing assay details

>hg38_dna range=chr2:113235183-113235270 5'pad=3 3'pad=3 strand=+ repeatMasking=none
Ccggcacccctacagcatccgccccttccgagcatgtcttccccgtcacagagaacttcatgttggcgcctccaaaagttgccggag

**Primer Set 2/2020**			**Score: 85<br>Quality: Medium**		
**Primer**	**Id**	**Sequence**	**Nt**	**Tm, °C**	**%GC**
PCR	PAX8-2/20-F1	TATGGGGTTTTGGGGTGGT	19	63.2	52.6
PCR	PAX8-2/20-R1	CCCCCTCTCTAACCTCAATCTCA	23	63.6	52.2
Sequencing	PAX8-2/20-S1	TTTGGGGTGGTGTAT	15	46.7	46.7
Target Polymorphisms	Position1, Position2, Position3, Position4, Position5				
Sequence to Analyze	T T TYGGTATT TTTATAGTAT TYGTTTTTTT YGAGTATGTT TTTTTYGTTA TAGAGAATTT TATGTTGGYG TTTTTAAAAG TTGT				
**Primer Pair**					
Amplicon length	161				
Score	94				
Amplicon %GC	34.8				

Details of post-mortem GTEx thyroid and whole blood samples

	**Number**
**Sex**	
Male	63
Female	23
**Age range (years)**	
20-29	5
30-39	6
40-49	15
50-59	33
60-69	24
70-79	3
**Death Classification**	
0	40
1	3
2	30
3	5
4	8

Death classification is based on the 4-point Hardy Scale:
0) Ventilator Case; All cases on a ventilator immediately before death.
1) Violent and fast death Deaths due to accident, blunt force trauma or suicide, terminal phase estimated at < 10 min.
2) Fast death of natural causes Sudden unexpected deaths of people who had been reasonably healthy, after a terminal phase estimated at < 1 hr (with sudden death from a myocardial infarction as a model cause of death for this category)
3) Intermediate death Death after a terminal phase of 1 to 24 hrs (not classifiable as 2 or 4); patients who were ill but death was unexpected
4) Slow death Death after a long illness, with a terminal phase longer than 1 day (commonly cancer or chronic pulmonary disease); deaths that are not unexpected
